# Impact of Different Exercise Modalities on the Human Gut Microbiome

**DOI:** 10.3390/sports9020014

**Published:** 2021-01-21

**Authors:** Dierdra Bycura, Anthony C. Santos, Arron Shiffer, Shari Kyman, Kyle Winfree, Jay Sutliffe, Talima Pearson, Derek Sonderegger, Emily Cope, J. Gregory Caporaso

**Affiliations:** 1 Department of Health Sciences, Northern Arizona University, Flagstaff, AZ 86011, USA; Anthony.Santos@nau.edu (A.C.S.); Jay.Sutliffe@nau.edu (J.S.); 2 Department of Biological Sciences, Northern Arizona University, Flagstaff, AZ 86011, USA; ams379@nau.edu (A.S.); Talima.Pearson@nau.edu (T.P.); Emily.Cope@nau.edu (E.C.); Greg.Caporaso@nau.edu (J.G.C.); 3 Center for Applied Microbiome Science, Pathogen and Microbiome Institute, Northern Arizona University, Flagstaff, AZ 86011, USA; skyman@tgen.org; 4 School of Informatics, Computing, and Cyber Systems, Northern Arizona University, Flagstaff, AZ 86011, USA; Kyle.Winfree@nau.edu; 5 Department of Mathematics and Statistics, Northern Arizona University, Flagstaff, AZ 86011, USA; Derek.Sonderegger@nau.edu

**Keywords:** gut, microbiome, exercise, resistance training, cardiorespiratory fitness

## Abstract

In this study we examined changes to the human gut microbiome resulting from an eight-week intervention of either cardiorespiratory exercise (CRE) or resistance training exercise (RTE). Twenty-eight subjects (21 F; aged 18–26) were recruited for our CRE study and 28 subjects (17 F; aged 18–33) were recruited for our RTE study. Fecal samples for gut microbiome profiling were collected twice weekly during the pre-intervention phase (three weeks), intervention phase (eight weeks), and post-intervention phase (three weeks). Pre/post VO_2max_, three repetition maximum (3RM), and body composition measurements were conducted. Heart rate ranges for CRE were determined by subjects’ initial VO_2max_ test. RTE weight ranges were established by subjects’ initial 3RM testing for squat, bench press, and bent-over row. Gut microbiota were profiled using 16S rRNA gene sequencing. Microbiome sequence data were analyzed with QIIME 2. CRE resulted in initial changes to the gut microbiome which were not sustained through or after the intervention period, while RTE resulted in no detectable changes to the gut microbiota. For both CRE and RTE, we observe some evidence that the baseline microbiome composition may be predictive of exercise gains. This work suggests that the human gut microbiome can change in response to a new exercise program, but the type of exercise likely impacts whether a change occurs. The changes observed in our CRE intervention resemble a disturbance to the microbiome, where an initial shift is observed followed by a return to the baseline state. More work is needed to understand how sustained changes to the microbiome occur, resulting in differences that have been reported in cross sectional studies of athletes and non-athletes.

## 1. Introduction

The human microbiota, the trillions of microbes living in and on the human body, play an important role in human health and disease. Humans begin life nearly sterile, and are rapidly colonized by microbes from their mother and their environment. As we age our microbiota composition is impacted by our diets, our habitats, and other aspects of our lifestyle. Many recent studies of the human gut microbiota have suggested that these microbial communities play a key role in human health, impacting metabolism [1], inflammation and mucosal barrier integrity [2,3], efficacy of drugs [4], and maintenance of host immune function [5]. There is growing interest in understanding how we can maintain a healthy microbial community or restore a dysbiotic one, and the relationship to overall health [6]. To achieve this, it is critical to understand factors that impact the microbiome and their respective effect sizes.

The effects of exercise on human health have been well studied [7]. Several research groups, primarily working in mice and humans, have recently explored how the vertebrate gut microbiome is impacted by exercise [8,9]. At least six studies have specifically explored the impact of exercise on the microbiome in humans through intervention studies with longitudinal microbiome profiling and these have shown differing results [10]. Allen et al. reported a 14-week study period with a six-week exercise intervention involving 18 obese and 14 lean individuals [11]. The composition of the microbiome in lean and obese individuals were different at the beginning of the study, but those differences were reduced following exercise intervention. Taniguchi et al. [12] performed a cross-over experiment where men between the ages of 62–76 were put on a five-week exercise program. The authors reported no observable changes in the gut microbiome composition or richness with exercise, though the abundances of *Clostridium difficile*, *Oscillospira*, and some functional (KEGG) pathways differed across exercise and control periods. Similarly, Munukka et al. [13] observed only modest changes in overall community composition following a six-week exercise intervention in 18 overweight women, and reported several taxa that appear to change in relative abundance with exercise. Cronin et al. [14] performed an eight-week combined aerobic and resistance training intervention study splitting participants (*N* = 90) into three groups: exercise-only, exercise with whey protein dietary supplement, and whey protein dietary supplement. They detected no significant changes in the microbiome due to exercise.

Exercise is often prescribed as a concomitant approach to treat metabolic diseases in general, and in diabetes management specifically. Liu et al. [15] conducted a 12-week intervention to determine the efficacy of exercise on the human gut microbiome as it relates to glucose homeostasis and insulin sensitivity in men with pre-diabetes. This exercise intervention consisted of a high intensity exercise training protocol that included both aerobic and strength training segments (three 10-min stations of treadmill, strength training and calisthenics, and stationary bicycling). The design of the exercise protocol incorporated a strength training aspect delivered in an aerobic modality. Therefore, the study findings were a result of aerobic training at a high intensity and not an anaerobic resistance training program per se. While cardiorespiratory fitness improvements were noted in the whole exercise group, the team identified high interpersonal variability in the alteration of fasting glucose, insulin, and glucose homeostasis and insulin sensitivity. Because of this, the investigators broke out the intervention group into responders and non-responders. A non-response was defined as a failure to demonstrate a decrease of homeostatic model assessment of insulin resistance (HOMA-IR; levels at 12-week against those at zero-week) that was greater than two-fold technical error from zero [16]. Examination of the microbiome in the full exercise group showed some significant changes in taxonomic composition and microbial functional pathways with exercise in responders. Furthermore, fecal transplants from exercise responders into obese mice reduced insulin resistance while fecal transplants from exercise non-responders did not.

Mailing et al. suggested that future inquiries should be designed to examine change in gut microbiota as a result of other modalities of exercise such as resistance training [8]. Energy metabolism during aerobic exercise differs from that of anaerobic metabolism and the metabolic pathway and fuel source utilized may impact human gut microbiome response. While Cronin et al. [14] incorporated limited resistance training within their weekly exercise intervention design, the focus of the exercise intervention was primarily aerobic, and the resistance training portion was not able to be analyzed independently. Previous studies did not separate resistance and aerobic training modalities, and had a very limited number of fecal samples which is needed to more closely examine change in microbial community over time as a result of exercise.

The aim of the present work was to examine potential change in the human gut microbiome as a result of different modes of exercise. Toward that end, we conducted two separate intervention studies with the same research design. The first, which we refer to as EXMP-CRE (Exercise Microbiome Project, Cardiorespiratory Exercise—CRE), focused on an eight-week cardiorespiratory exercise intervention. The second, which we refer to as EXMP-RTE (Exercise Microbiome Project, Resistance Training Exercise—RTE), focused on an eight-week hypertrophy-based resistance training exercise intervention. We hypothesized that an eight-week intervention of either modality of exercise would promote a change in the richness and composition of the human gut microbiota but that the gut microbiota would revert to its pre-intervention state after a wash out period, when study subjects resumed their sedentary behavior. We additionally explored whether an individual’s initial gut microbiota composition was predictive of their fitness gains in response to exercise intervention.

## 2. Materials and Methods

### 2.1. Subject Population and General Design

Students (18–33 years in age) enrolled at Northern Arizona University were recruited for participation in both studies via Institutional Review Board (IRB) approved recruitment fliers and emails (IRB approval EXMP-CRE: 899828-8 and EXMP-RTE: 1071194-5). The American Heart Association (AHA) and American College of Sports Medicine (ACSM) Health/Fitness Facility Preparticipation Screening Questionnaire were used to determine if subjects were healthy enough to participate in moderate to vigorous exercise prior to enrollment in either study. Exclusion criteria included screening subjects for historical factors that could impact their respective microbiome compositions, such as non-topical antibiotic use within the past six months, as well as current pregnancy status and whether they planned to become pregnant during either study period. Subjects were questioned about general dietary information (i.e., special dietary restrictions or food allergies) and home environment characteristics (i.e., whether pets are present or if they had recently completed a major geographical move). Additionally, subjects completed the International Physical Activity Questionnaire (IPAQ) Short Form (version 2.0). The Guidelines for Data Processing and Analysis of the IPAQ categorical scoring were used to determine subjects’ current level of physical activity. This information was not used as the exclusion criteria.

Subjects with AHA/ACSM Preparticipation Screening Questionnaire results indicating a need to seek medical consultation before engaging in exercise were excluded from participation in either study. All subjects provided written informed consent prior to taking part in either study. Upon inclusion into the studies, subjects were assigned a unique four- or five-digit alphanumeric code that was used for personal identification, exercise session attendance, and gut microbiome sample tracking. All subject data was de-identified prior to data analysis.

Study subjects provided two fecal swab samples per week over each 14-week study period. Fecal samples were self-collected by swabbing used toilet paper with sterile polyester swabs provided to subjects by study staff. Fecal samples were placed into containment tubes and submitted to study staff wrapped in an additional plastic bag. 

Subjects also completed an online per-sample questionnaire via Research Electronic Data Capture (REDCap™; Vanderbilt University, Nashville, TN, USA) that accompanied each fecal sample. Fecal samples were tracked using each subjects’ alphanumeric code and a barcode printed on each sample collection tube. Items queried in the per-sample questionnaire included whether or not subjects had significant changes to diet and exercise, antibiotic use, multivitamin and other nutritional supplementation, onset of illness and associated symptoms, and menstruation (see Spreadsheet S1, which lists questionnaire items). All subjects were instructed to maintain their normal dietary practices and physical activities throughout the length of both studies.

Both studies consisted of a three-week pre-intervention phase, an eight-week intervention phase, and a three-week post-intervention wash out phase (Table 1). We abbreviate these three phases as *Pre*, *Int*, and *Post*, respectively. Pre-intervention fitness and anthropometric measurements were conducted during the *Pre* phase. During the *Int* phase, all exercise sessions lasted 60 min and were attended three times per week. Exercise sessions ceased immediately prior to the *Post* phase of both studies, but subjects continued to submit twice weekly fecal samples. Post-intervention fitness and anthropometric measurements were conducted during the *Post* phase. No injuries were reported by subjects during either study period.

Both studies were conducted at an elevation of 2133 m which may have affected the results observed. However, all subjects lived at this elevation during the full durations of both studies, and performed both pre- and post-intervention testing at this elevation. 

As has been typical in other human microbiome exercise intervention studies [15,17], we did not directly control for diet. However, subjects in both studies were formally instructed to maintain their typical dietary practices during the entirety of each study period, and were asked to report any major deviations in the per-sample questionnaire that accompanied each fecal sample submission. Neither the CRE nor the RTE subjects reported major changes in diet on their per-sample questionnaires during either study period.

### 2.2. Procedures

#### 2.2.1. EXMP-CRE—Cardiorespiratory Fitness

The EXMP-CRE study sought to explore the effects of cardiorespiratory exercise on one’s gut microbial composition. During the eight-week *Int* phase, subjects attended three weekly cardiorespiratory exercise (CRE) sessions that lasted 60 min each. In accordance with ACSM guidelines for cardiorespiratory activity [18], subjects maintained an intensity between 60–90% maximal heart rate (HR_max_) during CRE sessions, as measured by Polar™ A300 HR monitors (Polar Electro; Kempele, Finland). Subjects’ heart rate ranges were established based on the maximal heart rate observed during pre-intervention maximal aerobic capacity (VO_2max_) treadmill testing. Subjects were frequently reminded by study staff to check their heart rate monitors during each CRE session to maintain the prescribed intensity range. To ensure exercise variability and maintain subjects’ engagement in the intervention, CRE intervention weeks consisted of two days of group cycling followed by one day of rotating CRE activity. These end-of-the-week sessions consisted of step aerobics, circuit training, non-contact kickboxing, stadium running, and other CRE activities. One certified group fitness instructor and one research assistant were present for all exercise sessions. Subjects repeated VO_2max_ treadmill testing during the three-week *Post* phase of the study.

#### 2.2.2. EXMP-RTE—Resistance Training

The EXMP-RTE study aimed to observe the effects of resistance training (RTE) on subjects’ gut microbial composition. Similar to EXMP-CRE, subjects attended three weekly exercise sessions that lasted 60 min each during the *Int* phase. RTE sessions implemented National Strength and Conditioning Association (NSCA) guidelines to improve muscular hypertrophy with subjects working at 70–85% one repetition maximum (1RM) over 3–6 sets of 6–12 total repetitions [19]. Subject weight ranges were based on pre-intervention three repetition maximum (3RM) testing for squat, bent-over barbell row, and bench press exercises in accordance with procedures outlined by the NSCA [19]. These specific lifts were selected to assess subjects’ muscular strength of the hips and legs, “pull” musculature of the posterior thorax and arms, and “push” musculature of the anterior thorax and arms, respectively. 

Sessions began with a warm-up period of dynamic stretching and full body movements and ended with static stretching focused on the major muscle groups employed during the workout. The three RTE sessions emphasized full, lower, and upper body exercises, respectively, and all included exercises stressing abdominal core musculature (see Spreadsheet S2, which provides subjects’ lifting cards used in the RTE intervention). Full body exercises included the three lifts performed during 3RM testing, as well as medicine-ball wall toss, hamstring curls using a stability ball, knee tucks using a stability ball, and Romanian deadlifts. Lower body exercises included sumo squats, glute bridges, traditional deadlifts, side lunges, and box jumps. Upper body exercises included bench press, bent-over barbell rows, reverse flies, pull-ups (assisted with resistance bands if needed), and mountain climbers.

All RTE sessions were supervised by one certified exercise physiologist and at least two research assistants. Subjects were provided with RTE session-specific lifting cards displaying pre-assigned weight and repetition values for each exercise. They were instructed to record their actual weight lifted and repetitions performed on these cards to allow study staff to better track and adjust weekly RTE intensities. Subjects’ weight values were collectively increased after the *Int* phase weeks 4 and 6 in the RTE study. Increases in weight were based on NSCA guidelines [19]. Subjects repeated 3RM testing during the *Post* phase of the study.

### 2.3. EXMP-CRE Measures

#### 2.3.1. Maximal Aerobic Capacity

The Bruce Protocol Treadmill Test was employed to establish subjects’ pre- and post-intervention VO_2max_ in mL·kg^−1^·min^−1^, respiratory exchange ratio (RER), and HR_max_ in beats per minute. The Bruce Protocol utilizes 3-min stages and begins at a speed of 2.7 km·h^−1^ and grade of 10% [20]. Stages incrementally increase both speed and grade to equate to increases of roughly three metabolic equivalents (METs) per stage. Subjects’ heart rates were recorded every 60 s and rate of perceived exertion (RPE) was recorded at the end of each completed stage. Test termination criteria included volitional fatigue, observation of subject heart rate at or above 90% of one’s age predicted HR_max_, RER of 1.10 or higher, and an RPE of 18 or higher (using Borg’s 6–20 scale) [18]. 

The COSMED K4b2 portable metabolic system (COSMED; Rome, Italy) was utilized to measure subjects’ heart rate, RER, and pulmonary gas exchange values. The K4b2 is a 1.2 kg research grade portable system worn on the backs of subjects for breath-by-breath analysis of pulmonary gas exchange. The system’s bidirectional digital turbine (28 mm diameter) was calibrated before the start of each testing date using a three-liter SensorMedics syringe (SensorMedics Corp.; Yorba Linda, CA, USA). The K4b2 gas analyzers were also calibrated before the start of each test date with ambient air and a proprietary gas mixture of 15% oxygen (O_2_) and 5% carbon-dioxide (CO_2_) composition. Heart rate was measured using a Polar™ heart rate monitor chest strap (Polar Electro; Kempele, Finland). Subjects’ VO_2max_ were recorded as the highest average value during one 15-s interval corresponding with one’s peak RER and HR values. 

Although not indicative of CRE performance gains, we did observe change in RER during post-intervention treadmill testing. RER values were grouped with other exercise intervention outcome measures due to its inclusion as criteria for establishing maximal aerobic capacity among subjects. Subsequent analyses of microbiome change accounting for significant difference in *Pre* and *Post* measures of RER do so as an observation. This is a reflection of this significant change and should not be regarded as causal or predictive of physiological changes that occurred in subjects from the eight-week CRE intervention.

#### 2.3.2. Ventilatory Threshold

To further investigate changes in aerobic capacity across the study period, subjects’ ventilatory thresholds (VT) were identified post hoc by V-slope plots [21] of O_2_ uptake (VO_2_) and CO_2_ output (VCO_2_) in mL·min^−1^ over pre- and post-test treadmill test times (s). Breath-by-breath sampling was averaged over 15 time-points in accordance with VO_2max_ procedures described above. The time-point where VCO_2_ crossed over VO_2_, indicating an increase in glucose over fatty acid metabolism, reliance on anaerobic metabolic pathways, and maxing out of aerobic pathways (i.e., nearing aerobic capacity), and its associated VO_2_ value in mL·min^−1^ were used to establish VT [21]. Subjects’ VT were confirmed by ventilatory equivalents plots of O_2_ (VE/VO_2_) and CO_2_ (VE/VCO_2_) by corroborating the time-point and mL·min^−1^ where VE/VO_2_ overtook VE/VCO_2_. After confirming all times of crossover, mL·min^−1^ values were converted to mL·kg^−1^·min^−1^ by dividing by subjects’ body weight in kg taken immediately prior to treadmill testing to align with relative VO_2max_ data.

#### 2.3.3. Anthropometric Measurements

Subjects’ height, weight, body mass index (BMI), fat-free mass (FFM), and percent body fat (%BF) were measured using a seca mBCA 514 Medical Body Composition Analyzer (seca; Hamburg, Germany) via bioelectrical impedance analysis (BIA). The seca 514 mBCA is a validated eight-electrode, segmental multifrequent device for measuring body composition in healthy adults of different ethnic populations. The test requires subjects to place each foot on two large electrodes while grasping two electrodes in each hand at waist height. Subjects were advised to avoid caffeine and alcohol intake within the 24 h time frame prior to testing. Study staff input subjects’ height, weight, age, gender, and ethnicity into the machine via touchscreen interface before the BIA commenced. Subjects’ deidentified data were then saved and sent to a desktop computer using a study-specific PIN. Subjects were tested during the *Pre* and *Post* phases.

### 2.4. EXMP-RTE Measures

#### 2.4.1. Submaximal Muscular Strength

Subjects performed pre- and post-intervention 3RM tests to estimate their 1RM weight; specifically, the greatest amount of weight that can be lifted with correct technique for only one repetition. Since many subjects did not have resistance training experience, 3RM tests were used to estimate maximal muscular strength to avoid musculoskeletal injury from overexertion or improper technique. Estimated 1RM (e1RM) values were derived by inputting subjects’ 3RM weight into the Brzycki equation [22] and were used as the central value upon which all training loads were calculated. All subjects were supervised during 3RM testing by multiple study staff and spotters.

Each subject began the 3RM tests with a warm-up set at a light resistance that easily allowed for 5–10 repetitions (reps). Following a 1-min rest period, resistance was increased by 4.5–9 kg or 5–10% for upper body exercise, and 13–18 kg or 10–20% for lower body exercises, to allow for a second warm-up set of 4–8 reps. Following a 2-min rest period, resistance was again increased by 4.5–9 kg or 5–10% for upper body exercise, and 13–18 kg or 10–20% for lower body exercises, and the subject performed a set of 4–6 reps at an estimated, conservative, near-maximal load. Following a 2–4 min rest period, the load was again increased by 4.5–9 kg or 5–10%, and the subject attempted to lift the weight for three reps. The subject then underwent another 2–4 min rest period.

If the subject was able to perform three reps without failure, the previous weight was again increased by 4.5–9 kg or 5–10%, and the subject again attempted to lift the weight for three reps after 2–4 min of rest. If the subject failed to perform three reps, the weight was decreased by 2.2–4.5 kg or 2.5–5%, and the subject again attempted to lift the weight for three reps after 2–4 min of rest. This process was repeated over 3–5 testing sets until the subject could complete no more, or less, than three reps with proper technique. The 3RM weight was then recorded and used to estimate the subject’s 1RM via the Brzycki equation. Subjects were tested on bench press, bent-over barbell row, and squat exercises to establish a baseline in push and pull upper body strength, and lower body strength, respectively. Prior to the pre-intervention 3RM test, all study subjects underwent a training session to ensure knowledge and practice of proper lifting technique during submaximal testing.

#### 2.4.2. Anthropometric Measurements

Subjects’ anthropometric measurements directly paralleled those of the EXMP-CRE study.

### 2.5. Statistical Analysis of Fitness and Anthropometric Measures

Descriptive statistics, paired-samples *t*-tests, and post hoc Pearson correlations evaluating relationships and differences in subjects’ fitness and anthropometric measurements were performed using IBM SPSS version 26.0 (SPSS Inc.; Chicago, IL, USA). Differences between study samples at baseline were assessed using independent *t*-tests. Our statistical significance threshold (alpha) was set at *p* ≤ 0.05 for all two-tailed statistical tests regarding exercise-related outcome variables. The fitness and anthropometric data presented in this study are available in Spreadsheet S3.

### 2.6. DNA Extraction and Microbiome Sequencing

Fecal samples were self-collected using BBL CultureSwabs (Becton, Dickinson, and Company; Sparks, MD, USA).

#### 2.6.1. EXMP-CRE

Total DNA was extracted from fecal swabs using DNeasy PowerSoil Kit (Qiagen, Hilden, Germany) using manufacturer’s protocol with one modification; to facilitate microbial lysis, swabs were incubated in lysis buffer for 10 min at 65 °C before mechanical lysis by vortexing in the PowerSoil Bead Solution. Resulting DNA samples were quantified on the Nanodrop 8000 Spectrophotometer (ThermoFisher; Waltham, MA, USA). For amplicon sequencing, barcoded 806R reverse primers and forward primer 515F were used to amplify the V4 region of the 16S rRNA gene [23,24]. Library preparation was done at the Pathogen and Microbiome Institute and sequencing was performed at the Translational Genomics Research Institute (TGen) Pathogen and Microbiome Division at Northern Arizona University. Each PCR reaction contained 2.5 µL of PCR buffer (TaKaRa, 10× concentration, 1× final), 1 µL of the Golay barcode tagged forward primer (10 µM concentration, 0.4 µM final), 1 µL of bovine serum albumin (ThermoFisher, 20 mg·mL^−1^ concentration, 0.56 mg·µL^−1^ final), 2 µL of dNTP mix (TaKaRa, 2.5 mM concentration, 200 µM final), 0.125 µL of HotStart ExTaq (TaKaRa, 5 U·µL^−1^, 0.625 U·µL^−1^ final), 1 µL reverse primer (10 µM concentration, 0.4 µM final), 16.375 µL PCR grade water (Sigma-Aldrich, St. Louis, MO, USA), and 1 µL template DNA. PCR conditions were as follows: 2 min at 98 °C for 1 cycle; 20 s at 98 °C, 30 s at 50 °C, and 45 s at 72 °C for 30 cycles; and 10 min at 72 °C for 1 cycle. Extraction blank negative controls were included in each extraction set and sequenced with the pool of fecal samples. A negative control for each barcoded primer was also run and visualized on a gel. If contamination was observed in the negative well, the sample was run with a new barcoded primer. PCR product was purified using AMPure XP for PCR Purification (Beckman Coulter; Indianapolis, IN, USA), quantified using Qubit dsDNA HS Assay Kit (ThermoFisher; Waltham, MA), and pooled at 25 ng per sample for sequencing on an Illumina MiSeq using MiSeq. Pooled amplicons were sequenced 251 × 12 × 251 using a MiSeq reagent kit v3 (Illumina; San Diego, CA, USA).

#### 2.6.2. EXMP-RTE

DNA extractions and microbiome sequencing were performed at the Environmental Sample Preparation and Sequencing Facility at the Argonne National Laboratory. Total DNA was extracted from fecal swabs using a MoBIO 96-well PowerSoil kit (Qiagen, Hilden, Germany) using the manufacturer’s protocol. Mechanical lysis was performed by vortexing samples in 96-well plates using the Powersoil Bead Solution. For amplicon sequencing, barcoded 806R reverse primers and forward primer 515F were used to amplify the V4 region of the 16S rRNA gene [23,24], PCR reactions contained 9.5 µL of MO BIO PCR Water (Certified DNA-Free), 12.5 µL of QuantaBio’s AccuStart II PCR ToughMix (2× concentration, 1× final), 1 µL Golay barcode tagged Forward Primer (5 µM concentration, 200 pM final), 1 µL Reverse Primer (5 µM concentration, 200 pM final), and 1 µL of template DNA. PCR conditions were as follows: 94 °C for 3 min to denature the DNA, with 35 cycles at 94 °C for 45 s, 50 °C for 60 s, and 72 °C for 90 s; with a final extension of 10 min at 72 °C to ensure complete amplification. Extraction blank negative controls were included in each extraction set and sequenced with the pool of fecal samples. PCR product was purified using AMPure XP for PCR Purification (Beckman Coulter; Indianapolis, IN, USA), quantified using Qubit dsDNA HS Assay Kit (ThermoFisher; Waltham, MA, USA), and pooled at equimolar concentrations for sequencing on an Illumina MiSeq using MiSeq. Pooled amplicons were sequenced 151 × 12 × 151 using a MiSeq reagent kit v3 (Illumina; San Diego, CA, USA).

### 2.7. Microbiome Bioinformatics

Microbiome bioinformatics was performed with QIIME 2 [25]. Our bioinformatics workflow was designed to facilitate the direct comparison of data from both of our studies. Denoising, paired-end read joining, and definition of amplicon sequence variants (ASVs) was performed using DADA2 [26] via the q2-dada2 QIIME 2 plugin. Because the reads from our two studies were of different lengths, we denoised using different trimming and truncation parameters for each (EXMP-CRE: trim_left_f/r = 0, trunc_len_f = 210, trunc_len_r = 160; EMXP-RTE: trim_left_f/r = 0, trunc_len_f/r = 151), and then merged the resulting joined paired-end ASV feature tables (which had the same start and end positions, so were fully overlapping). We reasoned that using more high-quality data would allow us to generate better taxonomic assignments downstream. We built a phylogenetic tree for computation of phylogenetic composition metrics using SEPP [27] in the q2-fragment-insertion plugin, which inserts ASV sequences into a tree generated from full-length sequences (the Greengenes tree by default). The feature table was filtered to remove any ASVs whose sequences could not be inserted into the reference tree with SEPP. Taxonomy was assigned using the naive Bayes classifier in q2-feature-classifier [28] trained on the Genome Taxonomy Database (GTDB) bacterial database release 89 [29]. Composition metrics were computed at an even sampling depth of 5000 sequences per sample.

Because denoising is generally performed with the same trimming and truncation parameters, we additionally experimented with that approach (trim_left_f/r = 0, trunc_len_f/r = 151), and with using the same trimming and truncation parameters using single-end reads only (trim_left_f = 0, trunc_len_f = 151). We compared weighted and unweighted UniFrac distances between samples using all three of these approaches, and found that the distance matrices were strongly correlated in all cases. We thus chose to proceed using our paired-end reads that used run-specific trimming and truncation parameters.

### 2.8. Statistical Analysis of Microbiome Measures

Longitudinal analysis of the microbiome was performed using the q2-longitudinal QIIME 2 plugin as well as custom visualization code provided in the project’s GitHub repository (https://github.com/caporaso-lab/exmp-paper1). Mann–Whitney U tests were performed to compare paired microbiome timepoints (e.g., distances from week 1) as implemented in SciPy. Associations between baseline microbiome composition and exercise performance changes were performed using ordinary least squares (OLS; as implemented in StatsModels), using PCoA axes and alpha diversity metrics as summary statistics for baseline microbiome composition. For each time point, we modeled performance changes from baseline as a function of the microbiome changes, age, and sex. Associations between microbiome change and exercise performance changes were performed using Spearman rank correlation between beta diversity distances and performance change measures. Adjustments for multiple comparisons across time points and diversity metrics was performed using the Benjamini–Hochberg method, as implemented in StatsModels.

## 3. Results

*Pre* phase fitness and microbiome metrics were utilized as subjects’ controls to compare pre-intervention and post-intervention measurements. A power analysis to determine sufficient sample sizes was not conducted. Our work, and a concurrent study by Allen et al. [11], were among the first such studies to examine microbiome changes associated with exercise. As the effect size of exercise on the microbiome was unknown, neither study conducted an a priori power analysis to calculate a necessary sample size. The Allen et al. [11] study had a sample size similar to ours (32 and 28, respectively). Changes in microbiome composition between *Pre* weeks on a per-individual basis provides subject-specific information on their typical gut microbiome dynamics (i.e., the amount of week-to-week variation that they typically experience).

### 3.1. Exercise Interventions

Independent samples t-tests revealed no significant differences in subjects’ age, *t*_30.52_ = −0.90, *p* = 0.38; body weight, *t*_40.45_ = −0.01, *p* = 0.99; BMI, *t*_46.00_ = 0.53, *p* = 0.60; FFM, *t*_32.88_ = −0.40, *p* = 0.69; and %BF, *t*_44.49_ = 0.19, *p* = 0.85, between both study samples at baseline. Average exercise session attendance rates for EXMP-CRE and EXMP-RTE were 89% and 86%, respectively. Subjects’ demographics in both EXMP studies are reported in Table 2. Outcome measures of both exercise interventions are presented in Table 3.

#### 3.1.1. EXMP-CRE—Cardiorespiratory Fitness

Subjects’ RER significantly increased among the total sample by an average of 0.07 units (*SD* = 0.13) following the CRE intervention, *t*_27_ = 2.80, *p* = 0.009, 95% CI [0.02, 0.12]. This difference was significant among female subjects (*M* = 0.09, *SD* = 0.13), *t*_20_ = 3.27, *p* = 0.004, 95% CI [0.028. 0.036]; but not male subjects (*M* = −0.01, *SD* = 0.08), *t*_6_ = −0.33, *p* = 0.75. Following the CRE intervention, time on treadmill during post-intervention VO_2max_ testing also significantly increased among the total study sample by an average of 51.22 s (*SD* = 76.17), *t*_27_ = 3.56, *p* = 0.001, 95% CI [21.68, 80.75]. A significant increase of 44.62 s (*SD* = 83.73) was also observed in female subjects, *t*_20_ = 2.44, *p* = 0.024, 95% CI [6.51, 82.73]; while a significant increase of 71.00 s (*SD* = 46.17) was observed in male subjects, *t*_6_ = 4.07, *p* = 0.007, 95% CI [28.30, 113.70]. VO_2max_ did not significantly differ pre- to post-intervention in the total sample (*M* = 0.03 mL·kg^−1^·min^−1^, *SD* = 2.91), *t*_27_ = 0.05, *p* = 0.96; female subjects (*M* = 0.06 mL·kg^−1^·min^−1^, *SD* = 2.74), *t*_20_ = 0.10, *p* = 0.93; or male subjects (*M* = −0.07 mL·kg^−1^·min^−1^, *SD* = 3.62), *t*_6_ = −0.05, *p* = 0.96. To investigate this further, Pearson correlation between change in body weight (kg) and change in relative VO_2max_ between *Pre* and *Post* measurements for the total sample were performed. Results suggest a strong negative linear relationship, *r*_26_ = −0.58, *p* = 0.001. This negative correlation with change in relative VO_2max_ was also evident with change in BMI (kg·m^−2^), *r*_26_ = −0.60, *p* = 0.001; and change in %BF, *r*_26_ = −0.49, *p* = 0.009. The eight-week CRE intervention seemed to have increased subjects’ post-intervention VO_2max_ test duration, but did not affect VO_2max_ itself.

Similar to Estaki et al. [30], subjects’ pre- and post-intervention VO_2max_ test values were sorted into established classifications for cardiorespiratory fitness (see Table, Spreadsheet S4, which displays subjects’ VO_2max_ classifications by sex) to further examine VO_2max_ changes on an individual basis. Categories were based on VO_2max_ classifications listed by Gibson, Wagner, and Heyward [31]. Overall, 15 subjects (54%) entered the intervention period with a “Poor” VO_2max_ classification, 10 entered with a “Fair” classification, and three entered with a “Good” classification. Most subjects (*n* = 22; 79%) maintained their pre-intervention VO_2max_ classification while five dropped one category. Only one subject improved one category, improving from “Poor” to “Fair.”

Change in body mass among subjects at post-test ranged between −4.10 and +4.70 kg. Pearson correlation further indicated a medium negative linear relationship between change in body weight (kg) and change in VT (mL·kg^−1^·min^−1^) between *Pre* and *Post* measurements, r_25_ = −0.40, *p* = 0.040. This negative correlation with change in VT was also apparent with change in BMI (kg·m^−2^), r_25_ = −0.44, *p* = 0.021; and change in %BF, r_25_ = −0.39, *p* = 0.045. These results suggest that VT values differed between VO_2max_ treadmill tests within subjects as a function of change in body mass. 

#### 3.1.2. EXMP-RTE–Resistance Training

Subjects’ 3RM and e1RM weights significantly increased across the squat, bench press, and bent-over row following the eight-week RTE intervention. In the full sample, subjects’ squat 3RM and e1RM weights increased by an average of 25.19 kg (*SD* = 14.11), *t*_27_ = 9.45, *p* < 0.001, 95% CI [19.72, 30.66]; and 26.70 kg (*SD* = 14.95), *t*_27_ = 9.45, *p* < 0.001, 95% CI [20.90, 32.50], respectively. For bench press, 3RM and e1RM weights increased an average of 7.69 kg (*SD* = 4.11), *t*_27_ = 9.90, *p* < 0.001, 95% CI [6.10, 9.29]; and 8.16 kg (*SD* = 4.36), *t*_27_ = 9.91, *p* < 0.001, 95% CI [6.47, 9.84], respectively. For bent-over row, 3RM and e1RM weights increased an average of 17.41 kg (*SD* = 5.92), *t*_27_ = 15.56, *p* < 0.001, 95% CI [15.12, 19.71]; and 18.46 kg (*SD* = 6.28), *t*_27_ = 15.56, *p* < 0.001, 95% CI [16.02, 20.89], respectively. FFM also significantly increased across the full sample following the RTE intervention by an average of 0.89 kg (*SD* = 2.16), *t*_26_ = 1.26, *p* = 0.042, 95% CI [0.04, 1.74]. Taken together, the RTE intervention proved effective in increasing mostly untrained subjects’ strength and lean body mass over 24 sessions in eight weeks. Further breakdown of strength gains by subjects’ sex is presented in Table 3.

In congruence with EXMP-CRE results, subjects were organized into relative strength (RS) categories pre- and post-intervention. RS was calculated by dividing subjects’ e1RM weights by body weight for the three lifts. Subjects were then categorized into RS classifications in accordance with percentile rankings and normative values published by Gibson et al. [31] and Hoffman [32] for bench press and squat, respectively. No normative values were found for bent-over row among this general, non-athletic, young-adult population. Overall, six subjects entered the intervention period with a “Well Below Average” bench press RS classification, three entered with a “Below Average” classification, three entered with an “Average” classification, eight entered with an “Above Average” classification, and eight entered with a “Well Above Average” classification. Twelve subjects experienced no change in RS for bench press, while 13 improved by one category and two improved by two categories. One subject improved by three categories, improving from “Below Average” to “Well Above Average.” For squat, 25 subjects entered the intervention period with a “Poor” RS classification and two entered with a “Fair” classification. One subject was classified as “Average” for squat relative strength prior to the intervention. Following the RTE intervention, 10 subjects experienced no change in squat RS, while five improved by one category, nine improved by two categories, one improved by three categories, and three improved by four categories (see Table, Spreadsheet S5, which displays subjects’ RS classifications by sex).

### 3.2. Cardiorespiratory Exercise Was Associated with Changes to Subjects’ Gut Microbiome

**Figure 1:**
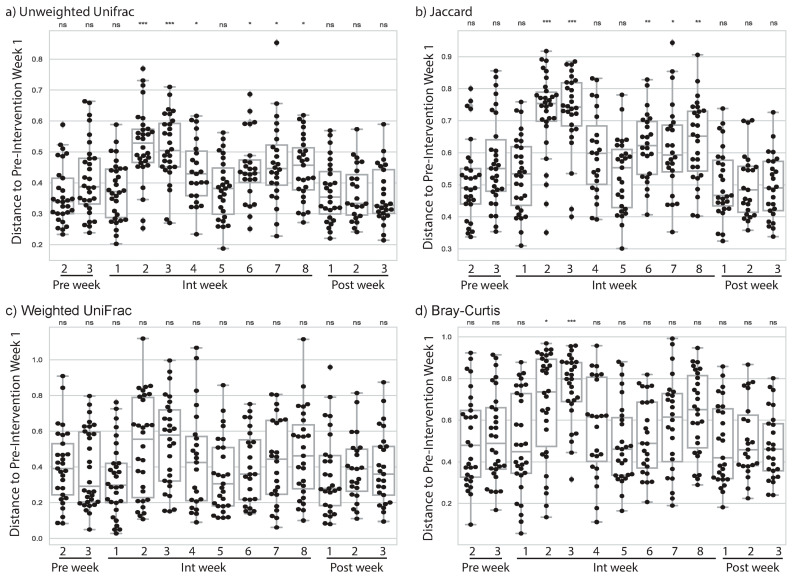
Microbiome change by week in EXMP-CRE. Microbiome dissimilarity to *Pre* phase week 1 (i.e., change since week 1) is reported using four different community dissimilarity metrics for *Pre* phase weeks 2–3, *Int* phase weeks 1–8, and *Post* phase weeks 1–3: (**a**) Unweighted UniFrac, (**b**) Jaccard distance, (**c**) weighted UniFrac distance, and (**d**) Bray–Curtis distance. Larger distances indicate more microbiome change from *Pre* phase week 1. Each box represents the distribution of microbiome change across all subjects. Significant change from week 1 was computed at each week using the Mann–Whitney U test. *ns* indicates no significant difference after correction for multiple comparisons; * indicates significant difference with *p* < 0.05 after correction for multiple comparisons; ** indicates significant difference with *p* < 0.01 after correction for multiple comparisons; and *** indicates significant difference with *p* < 0.001 after correction for multiple comparisons.

In EXMP-CRE we noticed a change in microbiome composition almost immediately after the initiation of the exercise program. In *Int* week 2, after a full week of exercise, subjects showed a significant change in unweighted UniFrac distance from *Pre* week 1 (Figure 1a), indicating a shift in their microbiome composition (*U* = 137.0; *p* = 3.23·10^−6^; FDR-corrected *p =* 4.20·10^−5^). This shift was also apparent when evaluated with another qualitative distance metric (Jaccard distance, Figure 1b), and a non-phylogenetic, quantitative distance metric (Bray-Curtis, Figure 1d) but not with a phylogenetic, quantitative distance metric (weighted UniFrac, Figure 1c). Since quantitative composition metrics downweight low abundance features and upweight high abundance features, and qualitative composition metrics highlight changes in the presence or absence of community members, taken together these results suggest that low abundance community members are either joining or leaving the community (or increasing or decreasing in abundance around our threshold of detection). 

Interestingly, the largest changes in the microbiome were observed in the second and third weeks of the *Int* phase, but around the fifth week of the *Int* phase, the magnitude of change decreased, with the change between *Pre* week 1 and *Int* week 5 not being significantly different from the change between *Pre* weeks 1 and 2 after adjustment for multiple comparisons. *Int* weeks 6, 7, and 8 were all more different from *Pre* week 1 than *Pre* week 2 was, but not as different as the early weeks in the *Int* phase. In *Post* phase week 1 the subjects were no longer more different from their *Pre* phase week 1 samples than they were in *Pre* phase week 2.

These results suggest that the addition of cardiovascular exercise prompts a change to the gut microbiome, that this change is most pronounced at the beginning of the program, but that it does not persist throughout or after completion of a short-term exercise intervention.

### 3.3. Resistance Training Was Not Associated with Changes to Subjects’ Gut Microbiome

**Figure 2:**
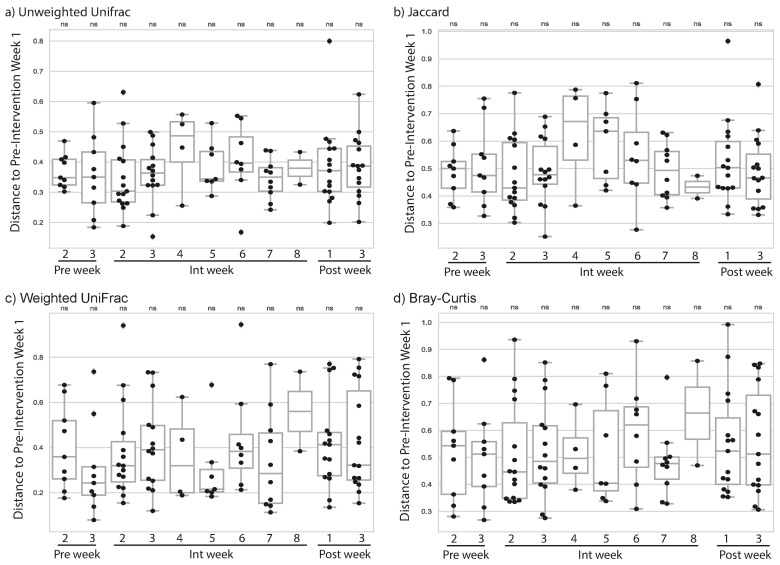
Microbiome change by week in EXMP-RTE. Microbiome dissimilarity to *Pre* phase week 1 (i.e., change since week 1) is reported using four different community dissimilarity metrics for *Pre* phase weeks 2–3, *Int* phase weeks 1–8, and *Post* phase weeks 1–3: (**a**) Unweighted UniFrac, (**b**) Jaccard distance, (**c**) weighted UniFrac distance, and (**d**) Bray–Curtis distance. Larger distances indicate more microbiome change from week 1. Each box represents the distribution of microbiome change across all subjects. Significant change from week 1 was computed at each week using the Mann–Whitney U test. *ns* indicates no significant difference after correction for multiple comparisons. Samples from fewer than five subjects were returned in *Int* week 2 and *Post* week 2, so we have excluded these data from the figure.

Unlike with our cardiorespiratory exercise intervention, we did not observe a change in microbiome composition with any of the four microbiome diversity metrics (Figure 2a–d). We generally had fewer subjects providing samples in EXMP-RTE, so this result may have been due to our smaller sample size. However, even in weeks when we had the largest number of samples and expected observed microbiome differences to be greatest based on our EXMP-CRE findings (e.g., *Int* week 2) we did not observe significant differences even before adjustment for multiple comparisons. Our findings therefore suggest that microbiome composition change is not a universal outcome of exercise intervention, but may be dependent on exercise modality.

### 3.4. Starting Microbiome State May Predict Magnitude of Change in CRE 

We next tested whether the microbiome composition pre-exercise was predictive of the change each individual might experience during the exercise intervention. As stated previously, change in RER was modeled as an observed significant difference between *Pre* and *Post* measurements as was time on treadmill. Exercise test performance is represented by subjects’ VO_2max_, VT, and time on treadmill (s). In EXMP-CRE, we evaluated OLS models when provided with Faith’s Phylogenetic composition, Shannon’s composition index, and Pileou’s Evenness index, and PCoA 1, 2, and 3 of unweighted and weighted UniFrac. We attempted to model change in RER and change in VO_2max_. For change in RER, the *Pre* week 1 microbiome, as defined by these summary metrics, was in congruence with significant change in RER as indicated during the post treadmill test (RER omnibus statistics: *F*_11, 16_ = 2.8, *p* = 0.031, *N* = 28) with Shannon’s diversity index, Pielou’s evenness index, and weighted UniFrac PCoA 3 as significant terms. For change in VO_2max_, the *Pre* week 1 microbiome nearly achieved statistical significance (VO_2max_: *F*_11, 16_ = 2.38, *p* = 0.056, *N* = 28) with the Shannon’s diversity index, Pielou’s evenness index, and weighted UniFrac PCoA 2 as significant terms. 

To interpret the association between PCoA axes and RER change, we computed Spearman correlation coefficients between all genera observed in EXMP-CRE and weighted UniFrac PCoA 3. This gives us an idea of the taxa that differ in the baseline gut microbiome composition across the individuals who demonstrated little to no change in RER versus some RER change post-intervention, and is similar to the analysis performed when generating microbiome composition biplots. The full results of this analysis are presented in Spreadsheet S6, which presents all correlation coefficients between genera in EXMP-CRE and weighted UniFrac PCoA 3 (*p*-values are not presented as we are not performing a statistical test, but rather ranking taxa based on their associations with the PCoA axes). 

These data suggest that the baseline gut microbiome of individuals who experience a higher RER *Post,* as measured during increased time on treadmill during Bruce Protocol VO_2max_ testing, are dominated by the genera *Prevotella*, *Romboutsia*, and *Dialister* (positive rho value between abundance and weighted UniFrac PCoA 3, with median abundance of at least 1), while the individuals who experience lower RER change *Post,* are dominated by the genera *Bacteroides*, *Bacteroides B*, and *Parabacteroides* (negative rho value between abundance and weighted UniFrac PCoA 3, with median abundance of at least 1). 

Because *Pre* weeks 2 and 3 also preceded exercise intervention, we additionally used these weeks to summarize the microbiome composition. In both of these cases the omnibus *p*-value was not significant, suggesting some noise in this signal. A recent study suggested that averaging microbiome composition across time points may be a useful approach to reduce noise in longitudinal studies [33], so we grouped *Pre* weeks 1–3 samples on a per subject basis by converting each feature count to its median count across the three *Pre* weeks of the study. When we did this and recomputed the microbiome metrics and our OLS model, we achieved a significant omnibus test result for VO_2max_ change (*F*_11, 16_ = 2.50, *p* = 0.047, *N* = 28) but not for RER change. In this analysis, weighted UniFrac PCoA 2 was the only significant term in the VO_2max_ model.

Full statistical results for all analyses described here can be found in Spreadsheet S7, which provides all results for all microbiome statistical analyses.

### 3.5. Starting Microbiome State Predicts Exercise Gains during Resistance Training

We additionally evaluated whether the starting microbiome state in EXMP-RTE predicted change in bench press, bent-over row, and squat. These specific variables were modeled according to their significance at *p* < 0.05, rather than using e1RM and 3RM for all three lifts. For change in 3RM squat, we observed that the *Pre* week 1 microbiome was predictive of exercise gains (*F*_6, 11_ = 5.16, *p* = 0.028, *n* = 18), with weighted UniFrac PCoA 2, weighted UniFrac PCoA 3, and the participant’s age as significant terms. None of the other tests resulted in a significant omnibus test result with any of the other three baseline weeks, or with the averaged baseline weeks (*n* = 18 in all tests), though e1RM bent-over row change was suggestive (*F*_6, 11_ = 2.56, *p* = 0.055, *n* = 18) when averaging the *Pre* weeks with unweighted UniFrac PCoA 1 as the only significant term. Again, this difference from our EXMP-CRE result may be due to the smaller sample size, or because the microbiome impacts, or is impacted differently, by different modes of exercise. 

To interpret the association between PCoA axes and 3RM squat change, we computed Spearman correlation coefficients between all genera observed in EXMP-RTE and weighted UniFrac PCoA 2 and 3. This gives us an idea of the taxa that contribute to the baseline gut microbiome composition for the individuals who experience low and high 3RM squat change over the course of the study, and is similar to the analysis performed when generating microbiome composition biplots. All correlation coefficients are presented in Spreadsheet S8, which presents correlation coefficients for weighted UniFrac PCoA 2, and Spreadsheet S9, which presents correlation coefficients for weighted UniFrac PCoA 3) for weighted UniFrac PCoA 2 and 3, respectively (*p*-values are not presented as we are not performing a statistical test, but rather ranking taxa based on their associations with the PCoA axes).

These data suggest that the baseline gut microbiome of individuals who experience high 3RM squat change are dominated by the Firmicutes genera *Ruminococcus* and unidentified Lachnospiraceae (positive rho value between abundance and weighted UniFrac PCoA 2, with median abundance of at least 1), and by the Firmicutes genera *Turicibacter* and *Clostridium* (positive rho value between abundance and weighted UniFrac PCoA 3, with median abundance of at least 1). The individuals who experience low 3RM squat change are dominated by the genera *Siccibacter*, *Bacteroides*, and *Bacteroides B* (negative rho value between abundance and weighted UniFrac PCoA 2, with median abundance of at least 1), and by the genera *Alistipes*, *Oscillibacter*, and *ER4* (family Oscillospiracea). Interestingly, while there is not much overlap between the taxa associated with higher exercise gains across EXMP-CRE and EXMP-RTE, two of the same genera (*Bacteroides* and *Bacteroides B*) are associated with lower gains in exercise performance in both studies.

Full statistical results for all analyses described here can be found in Spreadsheet S7, which provides all results for all microbiome statistical analyses.

### 3.6. Magnitude of Change in Microbiome Composition Is Not Correlated with Magnitude of Exercise Gains

We additionally evaluated whether the magnitude of microbiome change was associated with the magnitude of exercise performance change. To evaluate this, we tested for correlation between microbiome change at *Int* weeks 2 and 3 (when median microbiome changes were largest), and an individual’s change in VO_2max_ and RER for EXMP-CRE, and e1RM bench press, e1RM bent-over row, and 3RM squat for EXMP-RTE. For each study and performance metric, we performed a statistical test including all individuals in the study, as well as a sex-specific tests conducted on only males and only females in each study.

Results of statistical tests (Spearman rank correlations and scatter plots relating microbiome change to change in performance metrics) are available in Spreadsheet S10, which lists Spearman rank correlations and scatter plots relating microbiome changes to performance metrics. We did not observe any significant correlations in these tests, suggesting that individuals who have larger microbiome changes do not necessarily experience larger exercise performance gains. 

## 4. Discussion

Our initial questions were designed to examine whether an eight-week cardiorespiratory intervention (aerobic exercise) and/or an eight-week resistance training intervention (anaerobic exercise) altered the human gut microbiota. Secondly, we examined if initial gut microbiota profiles of subjects in either study predicted fitness adaptations. Changes in microbiota differed between exercise interventions, and the initial microbial community was predictive of fitness gains.

Microbiome changes due to aerobic, but not anaerobic, exercise may be due to differences in metabolic pathways specific to the exercise modalities. Internal physiological processes associated with CRE can divert blood flow away from the gastrointestinal (GI) tract, induce acute tissue hypoxia, decrease intestinal transit time, and increase absorptive capacity of the gut, further contributing to increased microbiota richness [30,34]. Exercise-induced increases to butyrate-producing taxa, and their ensuing production of butyrate, can lead to increased colonic epithelial cell proliferation, gut barrier integrity, and reduce inflammation throughout the gut [3,8]. It is clear that CRE exercise performed on a regular basis can positively impact human gut health, although the effects of intensity (e.g., high intensity interval training), duration, and initial microbiome status need further investigation. 

### 4.1. Change in Microbiome Associated with Cardiorespiratory Exercise

Exercise presents a potential stimulus for changes in the human microbiome richness and composition, though studies of microbiome composition with high temporal resolution have been lacking. Here we present our results of tracking the gut microbiome weekly over a period of 14 weeks, with individuals progressing through a three-week pre-exercise-intervention stage, an eight-week intervention period, and a three-week post-exercise-intervention phase. We find that by the second week of the exercise intervention (*Int* week 2) the individuals have experienced a compositional shift in their gut microbiome. This is evident when using qualitative distance metrics, such as unweighted UniFrac and Jaccard distance, but when using quantitative distance metrics, it is not observed (weighted UniFrac) or is less apparent (Bray–Curtis distance). By comparing different metrics across weeks, we can interpret the types of microbiome changes that occur. For example, as Bray–Curtis distance exhibits significant distances from *Pre* week 1 to *Int* weeks 2 and 3, but weighted UniFrac does not, this suggests changes in high abundance, but phylogenetically similar taxa. As unweighted UniFrac also exhibits significant distances from *Pre* week 1 to *Int* weeks 2 and 3, it suggests changes in the presence or absence of low abundance taxa that may be phylogenetically dissimilar. Together, these results suggest substantial changes in the gut microbiota in the first few weeks of exercise.

Because of the high temporal resolution of our studies relative to other human microbiome exercise intervention studies, we observe for the first time that exercise instigates large changes to the gut microbiome. This was apparent in all of the metrics that showed significant changes in our study. *Int* week 5 was not significantly different from *Pre* phase by any of our metrics, and *Int* weeks 6, 7, and 8 were always more similar to the *Pre* phase samples than the samples taken earlier in the exercise intervention. These data suggest an initial large change to the microbiome, but smaller differences from *Pre* phase once a few weeks have passed and the individual has settled into their new exercise regime. 

By all four of the metrics that we applied for comparing microbiome compositions we observed that, after the exercise intervention had completed, the individuals were no longer significantly different in their gut microbiome from their starting state. This suggests that the initial changes we see in response to exercise might resemble a disturbance to the gut microbiome, followed by a recovery to the pre-disturbance state as a homeostatic response to individual’s adaptation to the sustained exercise intervention. If true, this suggests that persistent microbiome differences between athletes and non-athletes, as have been reported from cross-sectional studies comparing athletes and non-athletes, may be the result of long-term lifestyle differences between these groups, including differences in diet and exercise practices, that cannot be quickly achieved.

### 4.2. Cardiorespiratory Fitness Adaptations

In our study sample for EXMP-CRE, we found a statistically significant increase in RER and time on treadmill (s) which may be related to increased ability to buffer lactate as a result of the eight-week intervention. RER values in excess of 1.00 relate to vigorous exercise intensities in which increased production of CO_2_ and hyperventilation result from buffering of excess hydrogen ions (H^+^) generated in anaerobic metabolic pathways within working musculature [21]. While increased RER at *Post* measurement is not indicative of cardiorespiratory fitness improvements, significant increases to RER and time on treadmill (s) during the stepped Bruce Protocol following the eight-week CRE intervention are worth noting.

We believe that the significant increases in subjects’ RER values and total treadmill test times (s) between *Pre* and *Post* VO_2max_ testing, without corresponding increases in VO_2max_ values, can be attributed to increased lactate buffering capacity resulting from the eight-week CRE intervention [35], although no direct measurements of lactate were performed during this study. Most subjects also entered the study with little experience in CRE, and the eight-week intervention period could have worked to normalize their subjective and physiological reactions to sustained cardiorespiratory work practiced during three weekly exercise sessions, resulting in their prolonged performance during the post-test Bruce treadmill Protocol.

Further, we attribute the lack of increase in relative VO_2max_ among most subjects to the relatively short duration of the CRE intervention and an increase in body weight among over half of subjects. Specifically, 15 subjects (54%) increased total body mass (ranging between 0.30–4.70 kg) during the study period. Post hoc Pearson correlations also support this interpretation, as increases in body weight, BMI, and %BF paralleled reductions to relative VO_2max_ in EXMP-CRE subjects. Similar results have been reported by Radovanović et al. [36] concerning BMI and VO_2max_ among adult men (*r* = −0.90, *p* < 0.001). Pribis et al. [37] also present negative correlations between both BMI and %BF and VO_2max_ among male (BMI: *r* = −0.33, *p* < 0.001; %BF: *r* = −0.49, *p* < 0.001) and female (BMI: *r* = −0.41, *p* < 0.001; %BF: *r* = −0.42, *p* < 0.001) college students. Additionally, changes in body weight might have affected improvements to VT following the eight-week CRE intervention. Most subjects (*n* = 24; 86%) also remained in the same VO_2max_ classification throughout the study period, with two subjects decreasing and two subjects increasing their VO_2max_ classifications. While we could not definitively pinpoint the cause of weight gain in our subjects, we postulate that self-report dietary intake was underreported in our college-aged population during the months of August–December in which the EXMP-CRE study took place. 

Intensity constraints of our CRE intervention (i.e., 60–90% HR_max_) were also more broadly defined than other similar studies. In comparison to Allen et al. [11], whose CRE intervention intensity incrementally progressed from 60% heart rate reserve (HRR) in weeks 1–3 to 75% HRR by week 6, our subjects were frequently reminded during exercise sessions to maintain a working intensity within our prescribed range. Subjects’ Polar™ heart rate monitors (Polar Electro; Kempele, Finland) were programmed to display working percentage of HR_max_ to better allow subjects to self-monitor during exercise sessions, and HR data showed an average working range of 70–85% HR_max_ over the intervention period. EXMP-CRE also provided subjects with greater variability in CRE exercise, with two weekly exercise sessions of group cycling and a third session of varying modality (e.g., step aerobics, bootcamp-style circuit training, non-contact kickboxing, stadium and track running, etc.) to more closely replicate how people might engage in exercise in a group setting rather than a controlled laboratory setting. 

It is possible that this variability might have worked against improvements to subjects’ maximal aerobic capacity in our eight-week intervention, as subjects in Allen et al.’s [11] study were provided either a cycle ergometer or treadmill during their six-week CRE intervention and saw an increase in VO_2max_ of at least 4 mL·kg^−1^·min^−1^ in both lean and obese groups. Allen et al. also reported a 100% compliance rate for their CRE intervention with mean subject ages of 25.10 (*SE* = 6.52) and 31.14 (*SE* = 8.57) years in lean and obese groups, respectively [11]. In comparison, the mean age of our undergraduate subjects was 20.54 (*SE* = 0.37) years, with 89% compliance over the CRE intervention. These differences in sample characteristics might also contribute to our lack of increase in relative VO_2max_ in EXMP-CRE.

Lastly, Allen et al. also acutely controlled for dietary consumption by asking subjects to follow a three-day food menu of only foods and drinks reported on a 7-day dietary recall prior to their three fecal sample collections [11]. They also instructed subjects to maintain overall dietary patterns over the entire study period, as did we. While our fecal sampling was far more extensive during both EXMP-CRE (*M* = 13.61, *SD* = 1.81, per subject) and EXMP-RTE (*M* = 7.44, *SD* = 1.33, per subject) studies, we only asked subjects to report substantial shifts in daily eating habits in online per-sample questionnaires accompanying each fecal sample taken, of which none did. We note that if dietary changes were large and unreported, this could potentially be a confounding variable that would offer an alternative explanation for the microbiome changes we observed in our cohort. Integrating food frequency questionnaires in future studies would help to isolate the cause of microbiome changes, though this is challenging to implement in studies involving volunteers due to the time these take to complete.

### 4.3. Resistance Training Associated Change in Microbiome

A gap in the exercise microbiome literature has been a comparison of different exercise modalities for their association with gut microbiome composition. In contrast to our findings in relation to CRE, we observed no significant changes during or after exercise intervention for individuals in our RTE exercise program. While our RTE study had the same number of subjects, in general we had lower adherence to microbiome sampling, and thus frequently had a lower sample size during individual weeks. As a result, it is possible that we do not observe differences in the microbiome with RTE exercise because we are under-powered. However, even in weeks where we observed large differences in microbiome composition during CRE, and where we had our largest sample size for RTE, we did not observe even weakly significant microbiome changes. We therefore conclude that the RTE either does not impact the microbiome, or does so with a much lower effect size than CRE. 

### 4.4. Resistance Training Fitness Adaptations

Significant increase in strength, measured across subjects’ 3RM for squat, bench press, and bent-over row, were related to neuromuscular adaptations, novelty of the RTE intervention to mostly untrained subjects, and the overall RTE program design implemented in EXMP-RTE. Initial improvements in muscular strength originate from neural adaptations across the central nervous system, and more distally at the neuromuscular junctions, that include greater motor unit recruitment, firing rates, and synchronization [19,38]. Subsequent strength gains are associated with increased muscle fiber hypertrophy and begin to occur after about 16 or more training sessions [19]. Because almost all subjects recruited for EXMP-RTE were untrained and novice weightlifters, we attribute most muscular strength gains to neuromuscular adaptations that occurred during the 24 RTE sessions of the eight-week intervention period. 

Untrained individuals can see increases in muscular strength of approximately 40% over training periods that ranged between four weeks and two years [39]. These increases were observed in the EXMP-RTE study with subjects increasing weights lifted in 3RM squat by an average of 40.46% (*SD* = 5.29, 95% CI [29.59, 51.33]), 3RM bench press by an average of 22.50% (*SD* = 3.10, 95% CI [16.12, 28.88]), and 3RM bent-over row by an average of 33.73% (*SD* = 2.66, 95% CI [28.27, 39.19]). Concurrent mean increases in FFM of 1.71% (*SD* = 0.69, 95% CI [0.30, 3.13]) in subjects might also relate to increased skeletal muscle fiber hypertrophy arising from the eight-week RTE intervention.

Additionally, untrained individuals adapt favorably in any RTE program and see a majority of strength increases within the first 4–8 weeks of training [38]. We expect that introduction to and participation in the eight-week intervention, which repeatedly trained all major muscle groups across a three-day weekly split of full body, upper body, and lower body exercises, contributed to the strength gains observed in our mostly untrained study sample. The intervention also stressed progressive overload by increasing weight lifted in each exercise during intervention weeks 4 and 6 in accordance with NSCA guidelines [19]. Increases in muscular strength are also personified using relative strength classifications for bench press and squat by Gibson et al. [31] and Hoffman [32], respectively (see Table, Spreadsheet S5).

### 4.5. Initial Microbiome Status and Subsequent Exercise Adaptations

Multiple recent studies suggest that an individual’s microbiome composition and richness might affect subsequent performance adaptations brought about by exercise [15,40]. For example, Chen et al. [40] demonstrated a positive correlation between endurance swimming time to exhaustion and higher microbiome richness in mice. Liu et al. [15] describe the development of a random forest regression model that relates improvement in glycemic control and following exercise based on the state of the microbiome prior to exercise. In this study, we constructed OLS models in an attempt to model direct measures of change in exercise performance metrics using summary statistics of the microbiome composition, including richness, evenness, and principal coordinates axes. We found that models could be developed to predict change in exercise performance. Of note, *Prevotella* abundance was associated with a higher RER during the post-treadmill test. *Prevotella* has been associated with plant-rich diets and is a producer of anti-inflammatory metabolites, short chain fatty acids, associated with gastrointestinal health [41]. This observation suggests that altering the gut microbiome either before or during exercise might allow for more effective exercise training, potentially helping individuals who are transitioning to an exercise program meet their performance improvement goals. While this is an exciting preliminary result, it is important to note that these models were developed on small cohorts, and some related approaches that we took toward modeling these data (described in Results) did not achieve statistical significance. Testing of this hypothesis in other, larger cohorts is essential. 

### 4.6. Next Steps in Understanding the Role of Exercise in Shaping the Microbiome

It is clear to us from this work and others that engaging in the ACSM minimum recommendations to improve cardiorespiratory fitness can impact the composition of the gut microbiome, although additional studies are essential to developing our understanding of that relationship. For example, it is highly likely that athletes harbor distinct microbiome compositions than less active individuals based on several cross-sectional studies [30,42], but at this point we do not know how long it takes for stable differences in the microbiome to emerge from exercise. It is also unclear what mechanisms drive microbiome change following the initiation of exercise, and if initial changes (as seen in the first few weeks of intervention in our cardiorespiratory study) have the potential to stabilize into the differences observed when comparing long-time athletes and non-athletes in cross-sectional studies. Our limited results suggest that changes are more likely to be a short-term response akin to a disturbance from which the individual’s microbiome returns to its pre-exercise state. Further, the degree to which exercise volume and/or intensity affect microbiome changes should also be examined in future research. If exercise proves to be a viable mechanism for introducing changes to an individual’s gut microbiome, for example to treat dysbiosis or impact disease-related phenotypes such as insulin sensitivity, these types of questions will need to be addressed. We suggest that in the next phase of exercise microbiome research, teams undertake the challenge of controlling or assessing diet in participants (e.g., by using validated food frequency questionnaires) to better differentiate the impacts of diet and exercise. We additionally suggest that dense temporal sampling is performed, for example weekly or even daily, to develop a better understanding of the microbiome dynamics that accompany the onset of exercise.

## Figures and Tables

**Table 1:** General study design.

Pre-Intervention Phase (Study Weeks 1–3; *Pre* Weeks 1–3)	Intervention Phase (Study Weeks 4–11; *Int* Weeks 1–8)	Post-Intervention Phase (Study Weeks 12–14; *Post* Weeks 1–3)
Initial subject meeting for informed consent, exercise sessions scheduling. Baseline fitness and anthropometric measurements. Subjects begin twice weekly fecal sample collection. ^a^	Exercise sessions performed 3x per week. Subjects continue twice weekly fecal sample collection.	Exercise sessions cease. Post-intervention fitness and anthropometric measurements. Subjects continue twice weekly fecal sample collection.

^a^ Average number of fecal samples collected per subject was 13.61 (SD = 1.81) over EXMP-CRE and 7.44 (SD = 1.33) over EXMP-RTE study periods.

**Table 2:** Subjects’ demographics and descriptive statistics.

	**EXMP-CRE**					
	**Total Sample (*N* = 28)**		**Female Subjects (*n* = 21)**		**Male Subjects (*n* = 7)**	
	**Pre**	**Post**	**Pre**	**Post**	**Pre**	**Post**
Age (year)	20.54 (1.93)		20.71 (1.88)		20.00 (2.16)	
Weight (kg)	67.83 (10.70)	68.14 (10.59)	66.22 (10.84)	66.57 (10.96)	72.63 (9.35)	72.86 (8.34)
BMI (kg·m^−2^)	24.41 (4.20)	24.55 (4.41)	24.54 (4.58)	24.72 (4.90)	24.04 (3.02)	24.06 (2.66)
FFM (kg)	48.71 (7.66)	48.85 (7.65)	45.28 (4.11)	45.43 (4.23)	59.01 (6.52)	59.11 (6.31)
%BF	27.57 (9.13)	27.68 (9.10)	30.62 (7.77)	30.71 (7.86)	18.43 (6.63)	18.57 (6.16)
	**EXMP-RTE**					
	**Total Sample (*N* = 28)**		**Female Subjects (*n* = 17)**		**Male Subjects (*n* = 11)**	
	**Pre**	**Post**	**Pre**	**Post**	**Pre**	**Post**
Age (year)	21.28 (3.85)		20.41 (3.34)		22.64 (4.34)	
Weight (kg)	67.72 (15.03)	68.32 (14.67)	61.58 (12.84)	62.08 (12.02)	77.20 (13.55)	77.97 (13.44)
BMI (kg·m^−2^)	23.77 (4.15)	23.97 (3.93)	23.24 (4.36)	23.43 (4.11)	24.59 (3.87)	24.81 (3.67)
FFM (kg)	49.58 (11.63)	50.47 (12.32) *	41.96 (5.15)	42.55 (5.12)	62.54 (6.89)	63.95 (8.48)
%BF	27.08 (8.10)	26.54 (8.35)	30.69 (6.49)	30.46 (6.00)	20.94 (6.92)	19.86 (7.67)

Values presented as mean (*SD*). * *p* < 0.05. Pre, pre-intervention; Post, post-intervention; BMI, body mass index; FFM, fat free mass; %BF, percent body fat.

**Table 3:** Exercise intervention outcome measures.

	**EXMP-CRE**								
	**Total Sample (*N* = 28)**			**Female Subjects (*n* = 21)**			**Male Subjects (*n* = 7)**		
	**Pre**	**Post**	**Δ**	**Pre**	**Post**	**Δ**	**Pre**	**Post**	**Δ**
VO_2max_ (mL·kg^−1^ ·min^−1^)	35.55 (6.48)	35.57 (5.88)	0.03 (2.91)	33.59 (5.64)	33.64 (5.08)	0.06 (2.74)	41.43 (5.38)	41.36 (4.23)	−0.07 (3.62)
RER	1.24 (0.12)	1.31 (0.09)	0.07 (0.13) **	1.21 (0.11)	1.31 (0.09)	0.09 (0.13) **	1.33 (0.11)	1.32 (0.11)	−0.01 (0.08)
Treadmill Test Time (s)	579.89 (114.78)	631.11 (98.49)	51.21 (76.17) **	550.48 (114.55)	595.10 (75.10)	44.62 (83.73) *	668.14 (58.54)	739.14 (81.84)	71.00 (46.17) **
	**EXMP-RTE**								
	**Total Sample (*N* = 28)**			**Female Subjects (*n* = 17)**			**Male Subjects (*n* = 11)**		
	**Pre**	**Post**	**Δ**	**Pre**	**Post**	**Δ**	**Pre**	**Post**	**Δ**
3RM Squat (kg)	71.28 (30.48)	96.47 (34.86)	25.19 (14.11) ***	56.83 (16.46)	77.78 (16.51)	20.95 (8.58) ***	93.60 (34.22)	125.36 (36.52)	31.75 (18.48) ***
Pred. 1RM Squat (kg)	75.54 (32.30)	102.24 (36.95)	26.70 (14.95) ***	60.23 (17.45)	82.43 (17.49)	22.20 (9.09) ***	99.20 (36.26)	132.86 (38.70)	33.64 (19.59) ***
3RM Bench Press (kg)	43.25 (22.20)	50.95 (22.88)	7.69 (4.11) ***	28.55 (6.32)	35.62 (6.20)	7.07 (3.75) ***	65.98 (18.19)	74.64 (18.30)	8.66 (4.63) ***
Pred. 1RM Bench Press (kg)	45.84 (23.52)	54.00 (24.24)	8.16 (4.36) ***	30.26 (6.69)	37.75 (6.57)	7.49 (3.98) ***	69.92 (19.27)	79.10 (19.39)	9.18 (4.90) ***
3RM Bent-Over Row (kg)	56.78 (23.36)	74.19 (25.78)	17.41 (5.92) ***	41.89 (7.61)	57.10 (8.49)	15.21 (4.73) ***	79.79 (20.53)	100.62 (20.34)	20.82 (6.15) ***
Pred. 1RM Bent-Over Row (kg)	60.18 (24.76)	78.63 (27.32)	18.46 (6.28) ***	44.40 (8.07)	60.52 (9.00)	16.12 (5.01) ***	84.56 (21.76)	106.63 (21.56)	22.07 (6.52) ***

Values presented as mean (*SD*). * *p* < 0.05. ** *p* < 0.01. *** *p* < 0.001. Pre, pre-intervention; Post, post-intervention; Δ, mean difference; VO_2max_, maximal aerobic capacity; RER, respiratory exchange ratio; 3RM, three repetition maximum; Pred., predicted; 1RM, one repetition maximum.

## Data Availability

Publicly available datasets were analyzed in this study. This data can be found in the NCBI Sequence Read Archive under BioProject ID PRJNA693579 (https://www.ncbi.nlm.nih.gov/bioproject/PRJNA693579).

## Supplementary Materials

The following are available online at https://www.mdpi.com/2075-4663/9/2/14/s1, Spreadsheet S1: Questionnaire completed by participants on sample submission; Spreadsheet S2: Upper body, lower body, and full body lifting cards used to track performance in EXMP-RTE; Spreadsheet S3: EXMP-CRE-RTE_DataFile; Spreadsheet S4: EXMP-CRE subjects’ VO_2max_ classifications by sex over study period; Spreadsheet S5: EXMP-RTE subjects’ relative strength categories by sex over study period; Spreadsheet S6: Correlation coefficients of microbial genera with weighted UniFrac PCoA 3 for EXMP-CRE; Spreadsheet S7: Ordinary least squares results from evaluations of relationship between baseline microbiome and exercise performance change; Spreadsheet S8: Correlation coefficients of microbial genera with weighted UniFrac PCoA 2 for EXMP-RTE; Spreadsheet S9: Correlation coefficients of microbial genera with weighted UniFrac PCoA 3 for EXMP-RTE; Spreadsheet S10: Correlations test results and scatterplots illustrating relationships between exercise performance change and microbiome change.
