# Supplementary material for: Impact of Different Exercise Modalities on the Human Gut Microbiome
Source: Sports (Basel). 2021 Jan 21;9(2):14. doi: 10.3390/sports9020014 (PMC7909775; doi:10.3390/sports9020014)
Supplement: Supplementary file 1 [file sports-09-00014-s001.zip › Supplementary Materials/Spreadsheet S1.pdf]

Per Sample Questionnaire

Record ID ________________________________

Sampling date and time ________________________________
(Please use the following format: dd/mm/yy 00:00 AM/PM)

Sampling bar code ID ________________________________
(Enter the barcode ID from the barcode on the sample tube.)

Your current weight in pounds. ________________________________
(This is an important part of the study so please be as accurate as possible. Weight in pounds. No units necessary.)

Has your diet changed significantly since the last sampling event?
- ◯ Yes
- ◯ No

(It is important that diet remain consistent for the entire study period. Please report diet inconsistencies here.)

In what way has your diet changed significantly?

____
(Here you would describe eating foods you would not have normally eaten. List changes to diet that have significantly increased or decreased caloric count or significant changes in the types of food you have been eating.)

Have you begun taking non-antibiotic prescriptions, daily multivitamins, or other nutritional supplements since the last sampling event?
- ◯ Antibiotics
- ◯ Non-antibiotic prescriptions
- ◯ Supplements
- ◯ None of the above

Please list what supplements, antibiotics, or prescriptions you have begun taking, the dose and purpose for the supplement.

____

Has your exercise routine changed significantly since the last sampling period?
- ◯ Yes
- ◯ No

(It is important that exercise routine (with the exception of the exercise being added as part of this study) remain consistent for the entire study period. Please report exercise inconsistencies here. Here you would describe physical activity you would not normally have performed or a failure to perform physical activity that you normally would have.)

In what way has your exercise routine changed significantly?

____
(Here you would describe physical activity you would not normally have performed or a failure to perform physical activity that you normally would have.)

If you have been sick since the last sampling event please check all symptoms that apply.

- ☐ Stomach ache
- ☐ Fever
- ☐ Vomiting
- ☐ Cold sweats
- ☐ Body aches
- ☐ Headache
- ☐ Runny nose
- ☐ Congestion
- ☐ Coughing
- ☐ Other

Are you female?

- ◯ Yes
- ◯ No

Are you currently or have you menstruated since the last sampling day?

- ◯ Yes, I am currently menstruating
- ◯ Yes, I menstruated since the last sampling day but am not currently
- ◯ No, I am not and have not menstruated since the last sampling day
- ◯ No, I am taking birth control that does not permit me to menstruate
- ◯ No, I am pregnant or suspect that I may be pregnant.

(Menstruation has been shown to significantly affect microbiome composition.)

Have there been any other changes in your routine activities that you think could influence the microbial communities associated with your various body habitats?

____

((e.g. quit smoking, moved residence, got flu vaccine etc.))
