# Supplementary material for: Impact of Different Exercise Modalities on the Human Gut Microbiome
Source: Sports (Basel). 2021 Jan 21;9(2):14. doi: 10.3390/sports9020014 (PMC7909775; doi:10.3390/sports9020014)
Supplement: Supplementary file 1 [file sports-09-00014-s001.zip › Supplementary Materials/Spreadsheet S10.pdf]

project	distance	performance metric	Spearman rho	Spearman p-value
exmp1	unweighted UniFrac distance (week 1 to 5.0)	RER-change	0.223648123	0.262118472
exmp1	unweighted UniFrac distance (week 1 to 5.0)	VO2max-change	0.384004884	0.047985996
exmp1	weighted UniFrac distance (week 1 to 5.0)	RER-change	0.022609237	0.910874146
exmp1	weighted UniFrac distance (week 1 to 5.0)	VO2max-change	0.357753358	0.066928415
exmp1	Bray-Curtis distance (week 1 to 5.0)	RER-change	0.141154963	0.482501569
exmp1	Bray-Curtis distance (week 1 to 5.0)	VO2max-change	0.36996337	0.057506101
exmp1	Jaccard distance (week 1 to 5.0)	RER-change	0.048579305	0.809847438
exmp1	Jaccard distance (week 1 to 5.0)	VO2max-change	0.371794872	0.056186897

sample size	Spearman q-value
27	0.703553346
27	0.325244093
27	0.963842003
27	0.332882987
27	0.861411527
27	0.332882987
27	0.963842003
27	0.332882987

project	distance	performance metric	Spearman rho	Spearman p-value
exmp1	unweighted UniFrac distance (week 1 to 6.0)	RER-change	-0.1964409	0.336145609
exmp1	unweighted UniFrac distance (week 1 to 6.0)	VO2max-change	0.056410256	0.784308648
exmp1	weighted UniFrac distance (week 1 to 6.0)	RER-change	-0.17830263	0.383498543
exmp1	weighted UniFrac distance (week 1 to 6.0)	VO2max-change	-0.17538462	0.391456855
exmp1	Bray-Curtis distance (week 1 to 6.0)	RER-change	-0.07357978	0.720928979
exmp1	Bray-Curtis distance (week 1 to 6.0)	VO2max-change	-0.18837607	0.356747017
exmp1	Jaccard distance (week 1 to 6.0)	RER-change	-0.12183442	0.553248967
exmp1	Jaccard distance (week 1 to 6.0)	VO2max-change	0.016752137	0.935263896

sample size	Spearman q-value
26	0.96227905
26	0.97826577
26	0.96227905
26	0.96227905
26	0.97826577
26	0.96227905
26	0.96227905
26	0.97826577

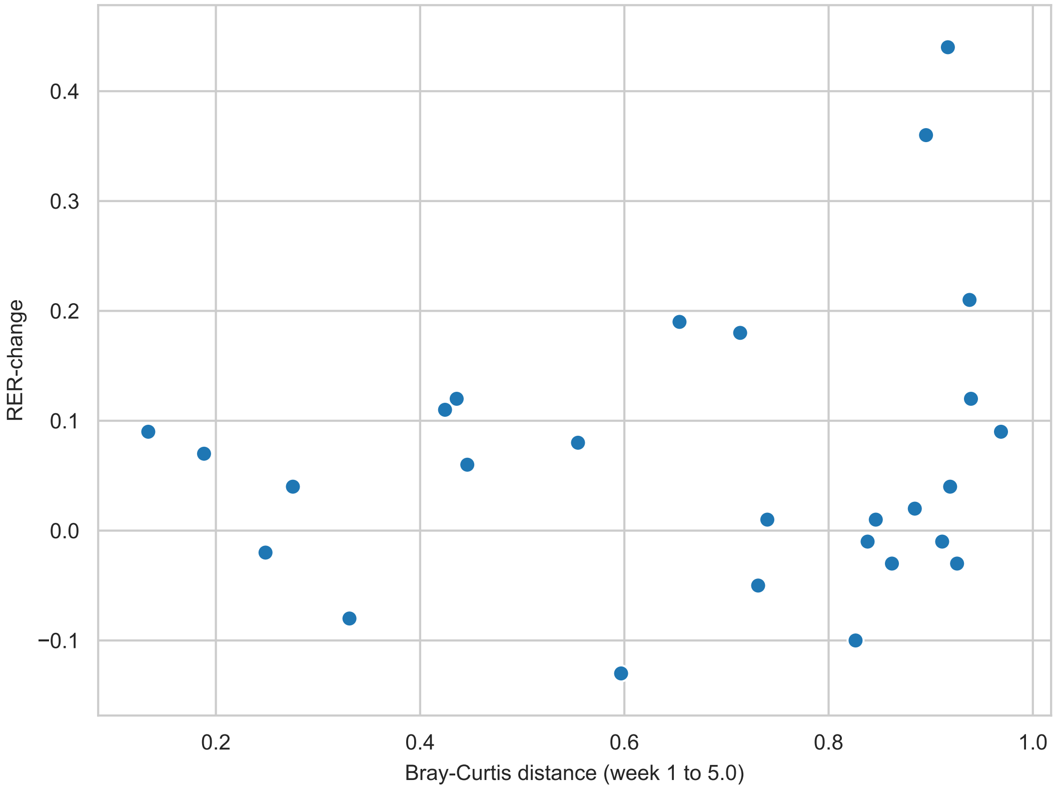
0.4
0.3
0.2
0.1
0.0
−0.1
RER-change
0.2
0.4
0.6
0.8
1.0
Bray-Curtis distance (week 1 to 5.0)

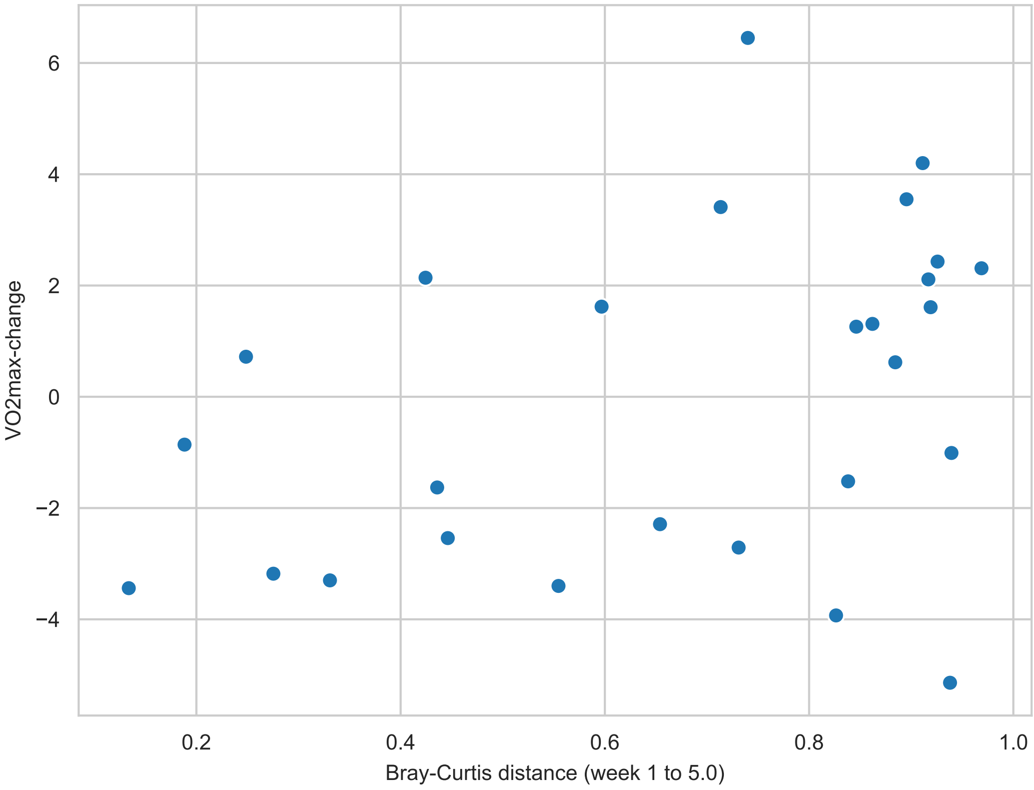
VO2max-change
Bray-Curtis distance (week 1 to 5.0)
6
4
2
0
−2
−4
0.2
0.4
0.6
0.8
1.0

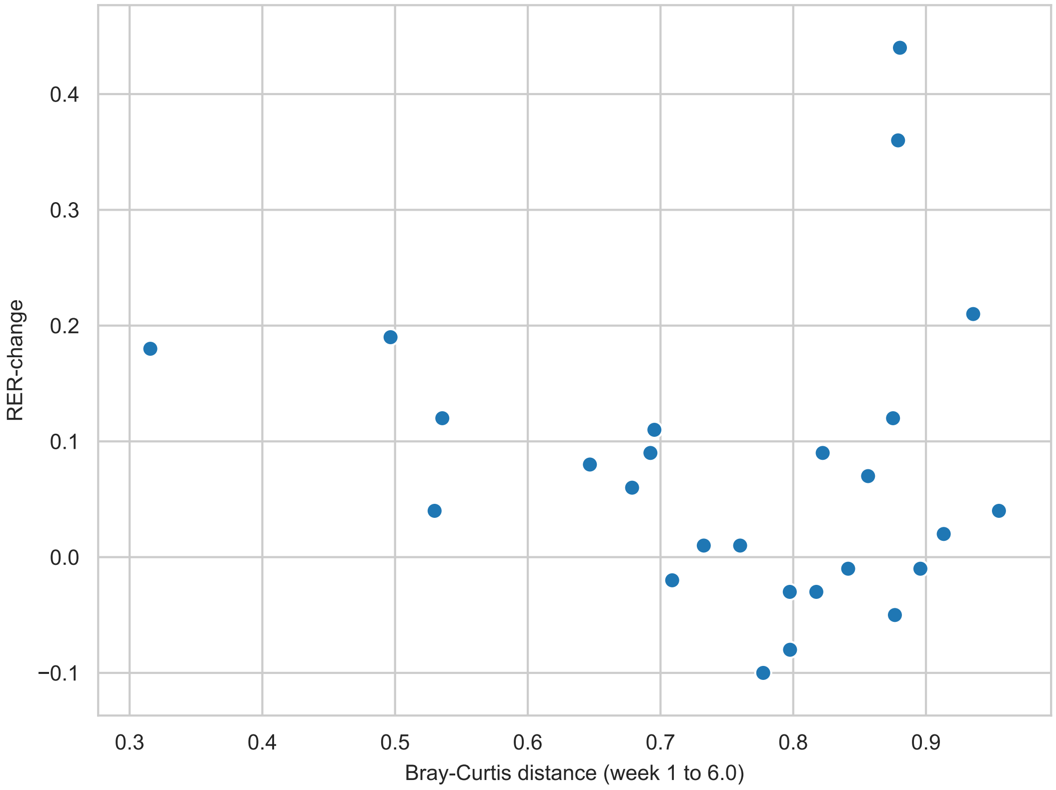

0.4
0.3
0.2
0.1
0.0
−0.1
RER-change
0.3
0.4
0.5
0.6
0.7
0.8
0.9
Bray-Curtis distance (week 1 to 6.0)

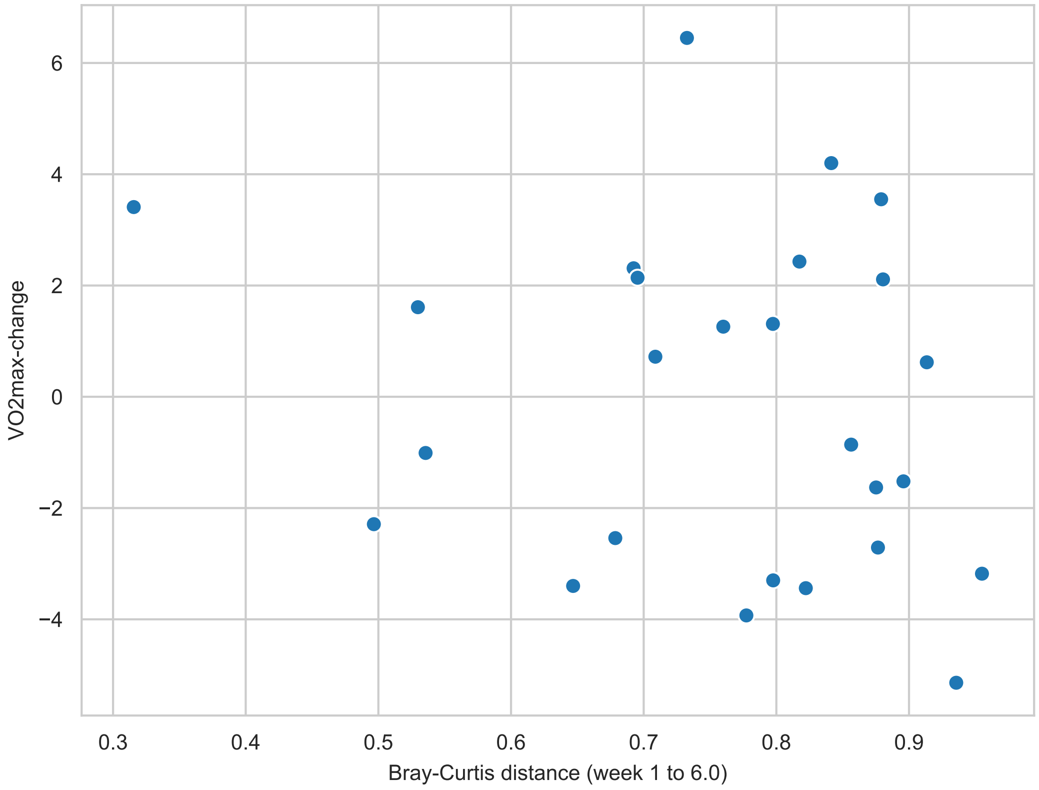
6
4
2
0
−2
−4
VO2max-change
0.3
0.4
0.5
0.6
0.7
0.8
0.9
Bray-Curtis distance (week 1 to 6.0)

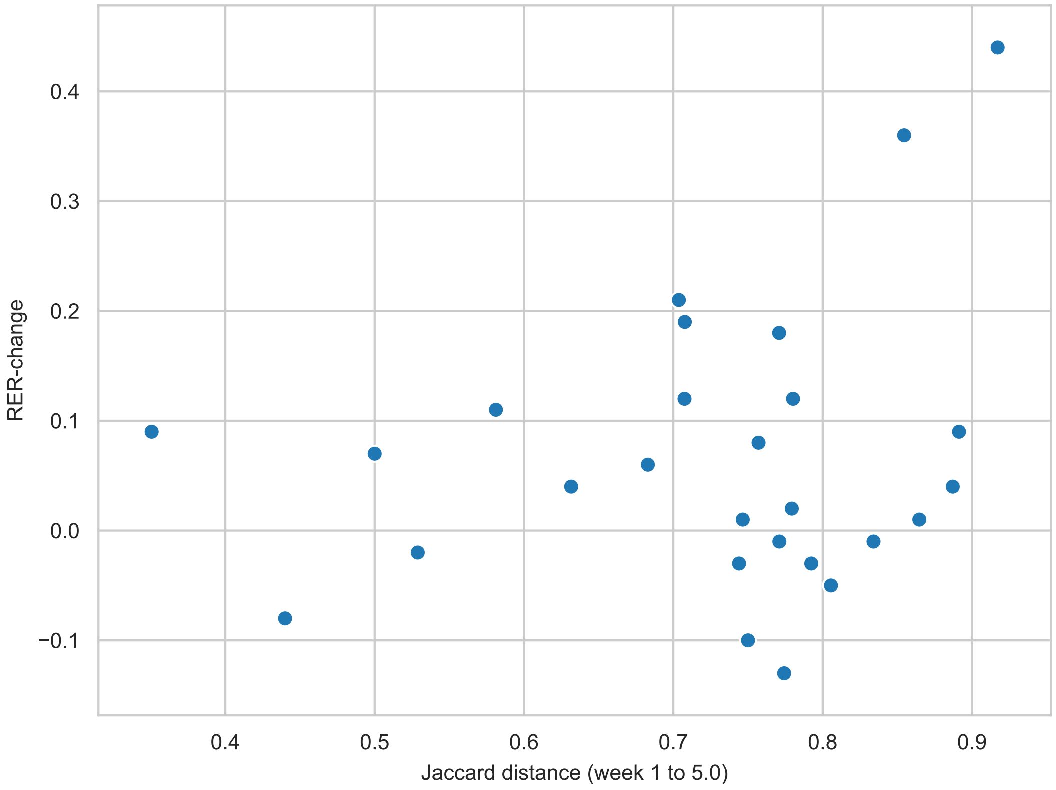

RER-change
0.4
0.3
0.2
0.1
0.0
−0.1
0.4
0.5
0.6
0.7
0.8
0.9
Jaccard distance (week 1 to 5.0)

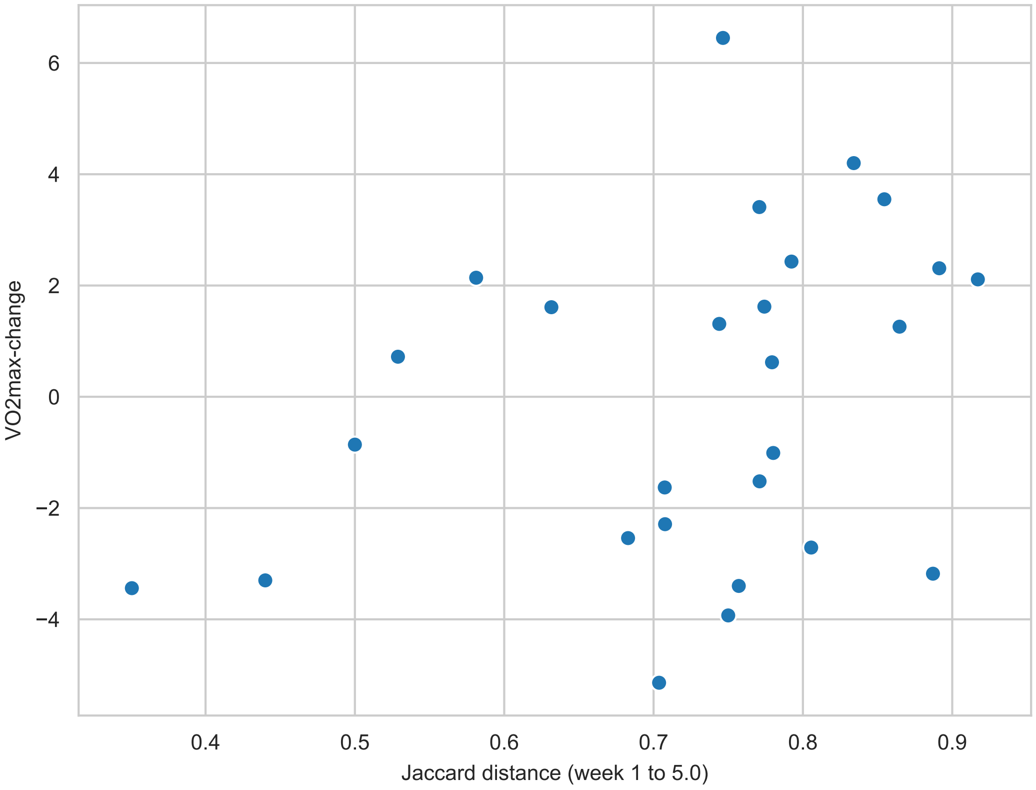

6
4
2
0
−2
−4
VO2max-change
0.4
0.5
0.6
0.7
0.8
0.9
Jaccard distance (week 1 to 5.0)

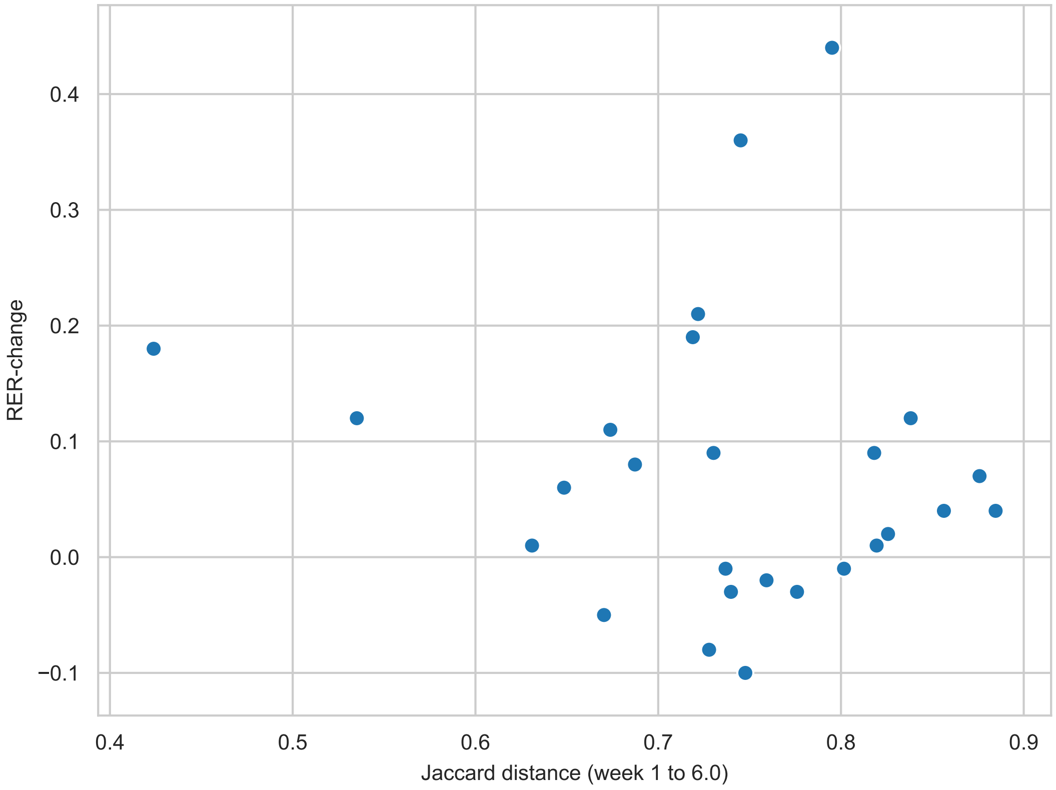

0.4
0.3
0.2
0.1
0.0
−0.1
RER-change
0.4
0.5
0.6
0.7
0.8
0.9
Jaccard distance (week 1 to 6.0)

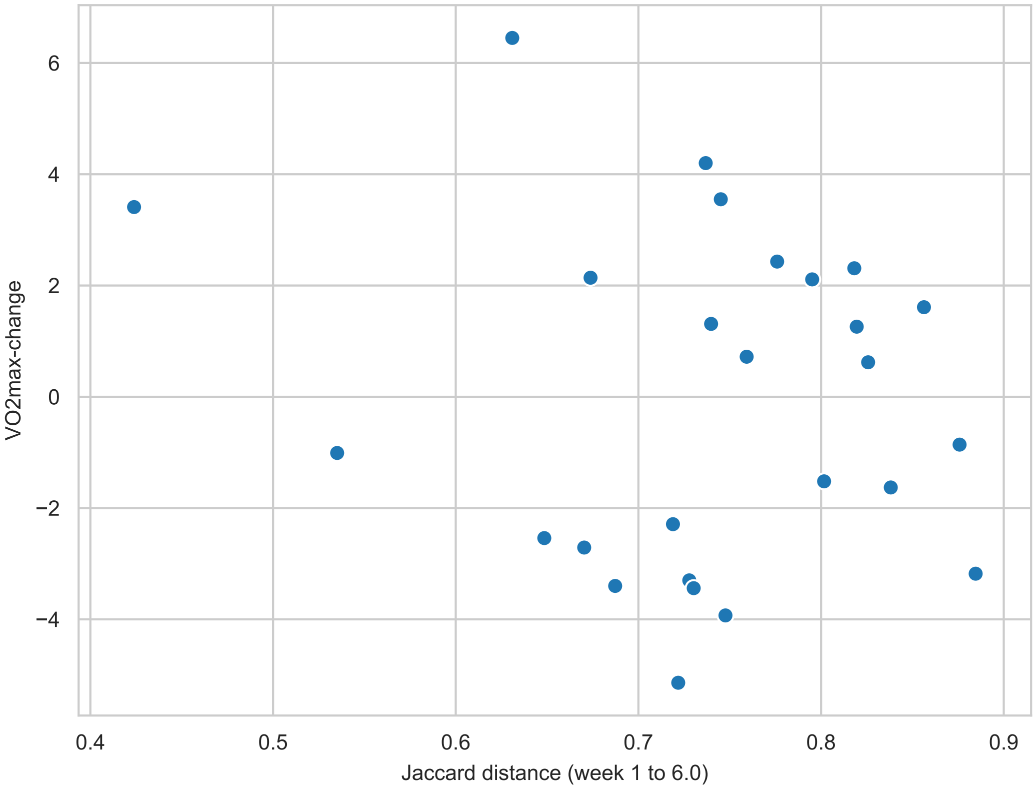
6
4
2
0
−2
−4
VO2max-change
0.4
0.5
0.6
0.7
0.8
0.9
Jaccard distance (week 1 to 6.0)

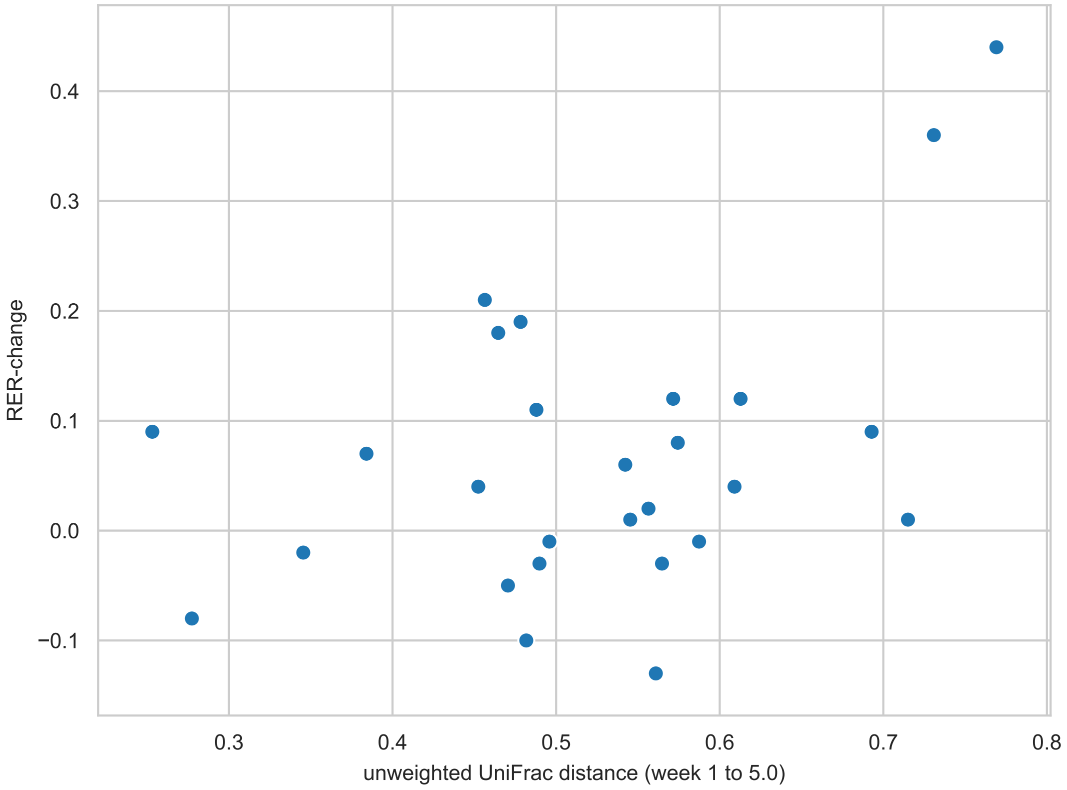

0.4
0.3
0.2
0.1
0.0
−0.1
RER-change
0.3
0.4
0.5
0.6
0.7
0.8
unweighted UniFrac distance (week 1 to 5.0)

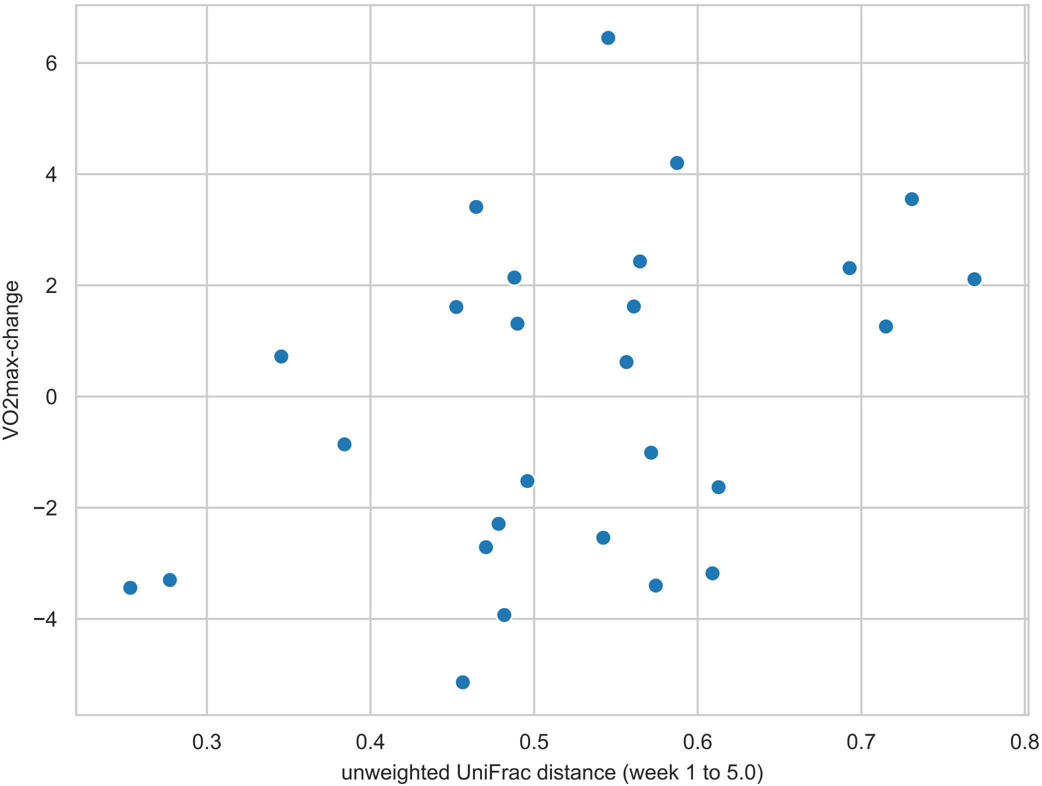
6
4
2
0
−2
−4
VO2max-change
0.3
0.4
0.5
0.6
0.7
0.8
unweighted UniFrac distance (week 1 to 5.0)

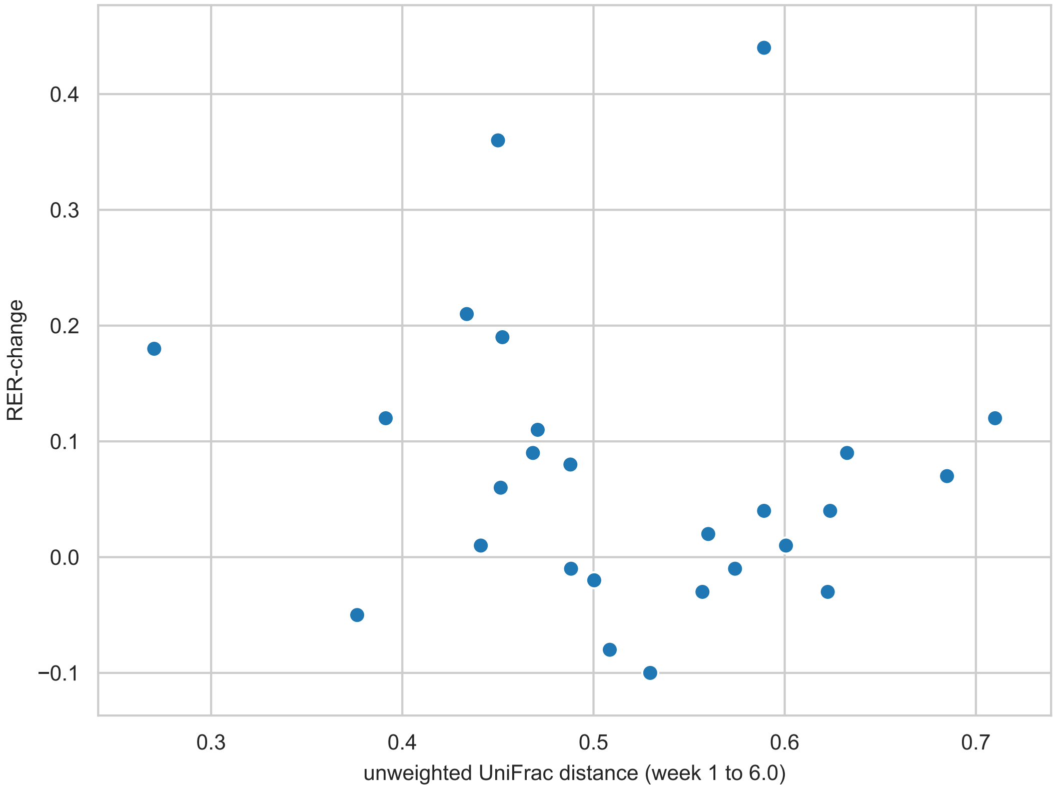

RER-change
0.4
0.3
0.2
0.1
0.0
−0.1
0.3
0.4
0.5
0.6
0.7
unweighted UniFrac distance (week 1 to 6.0)

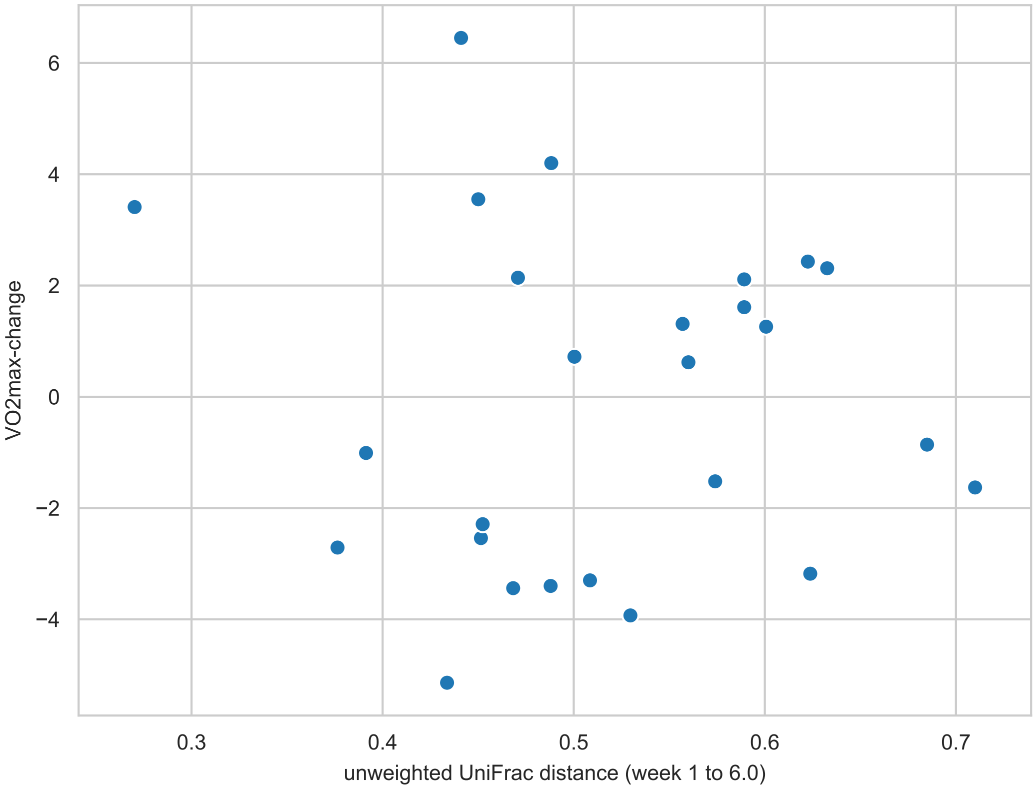

6
4
2
0
−2
−4
VO2max-change
0.3
0.4
0.5
0.6
0.7
unweighted UniFrac distance (week 1 to 6.0)

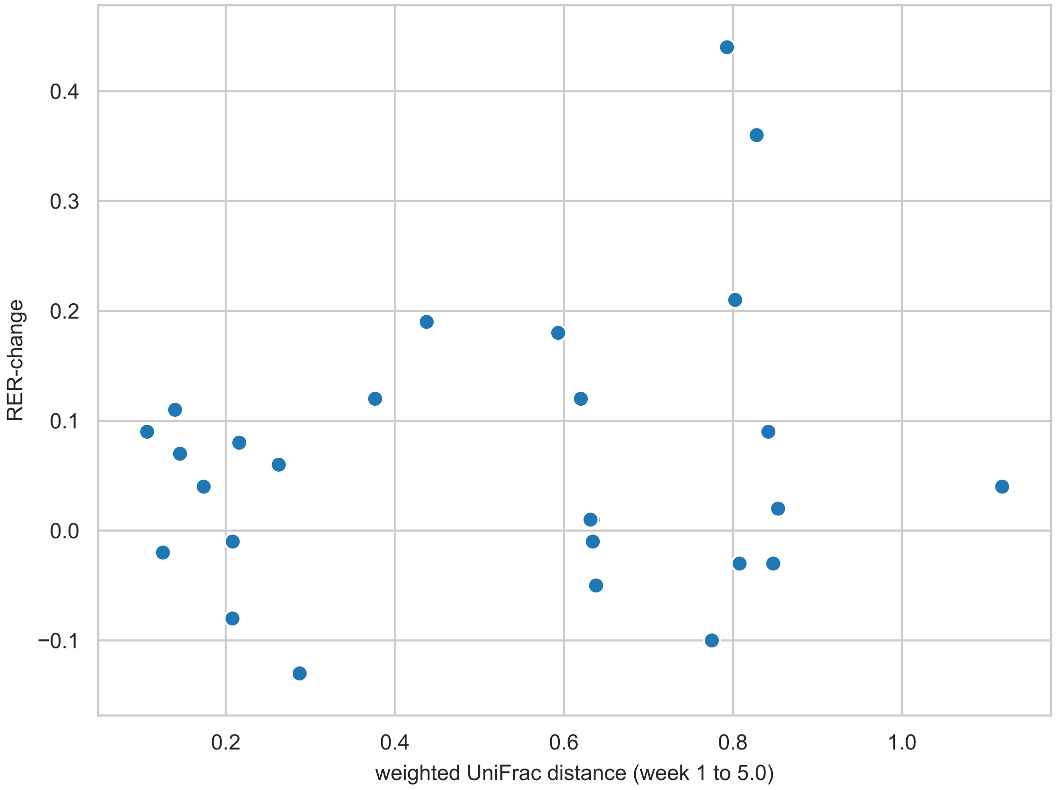

0.4
0.3
0.2
0.1
0.0
−0.1
RER-change
0.2
0.4
0.6
0.8
1.0
weighted UniFrac distance (week 1 to 5.0)

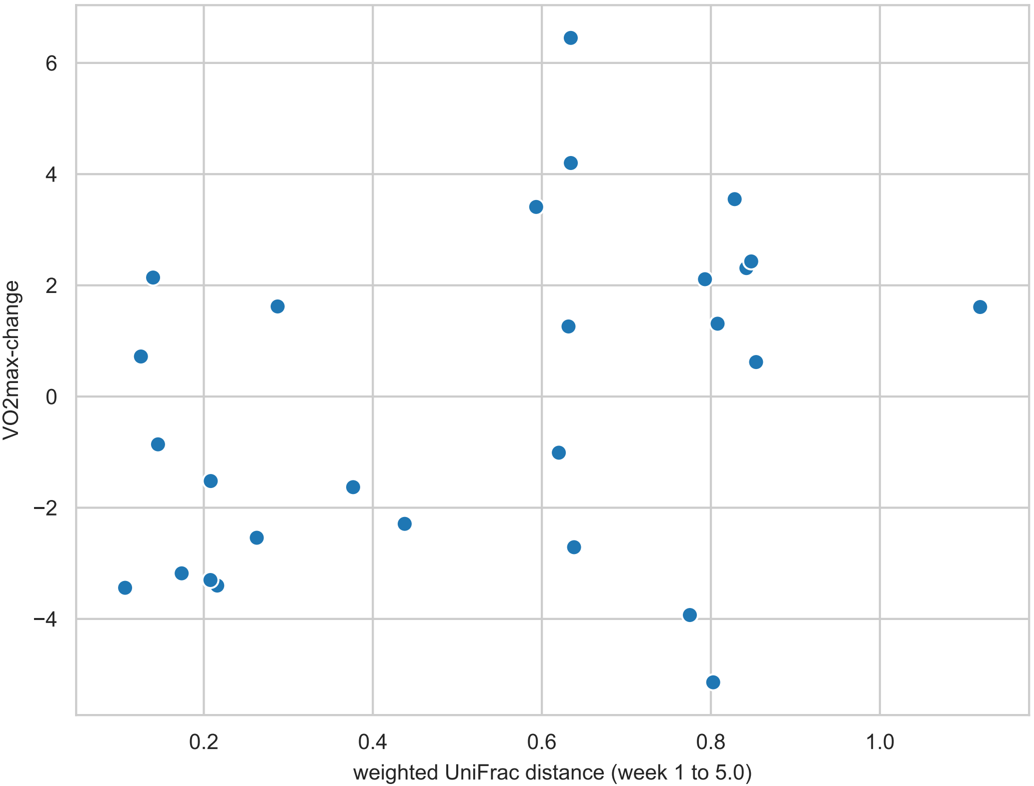

6
4
2
0
−2
−4
VO2max-change
0.2
0.4
0.6
0.8
1.0
weighted UniFrac distance (week 1 to 5.0)

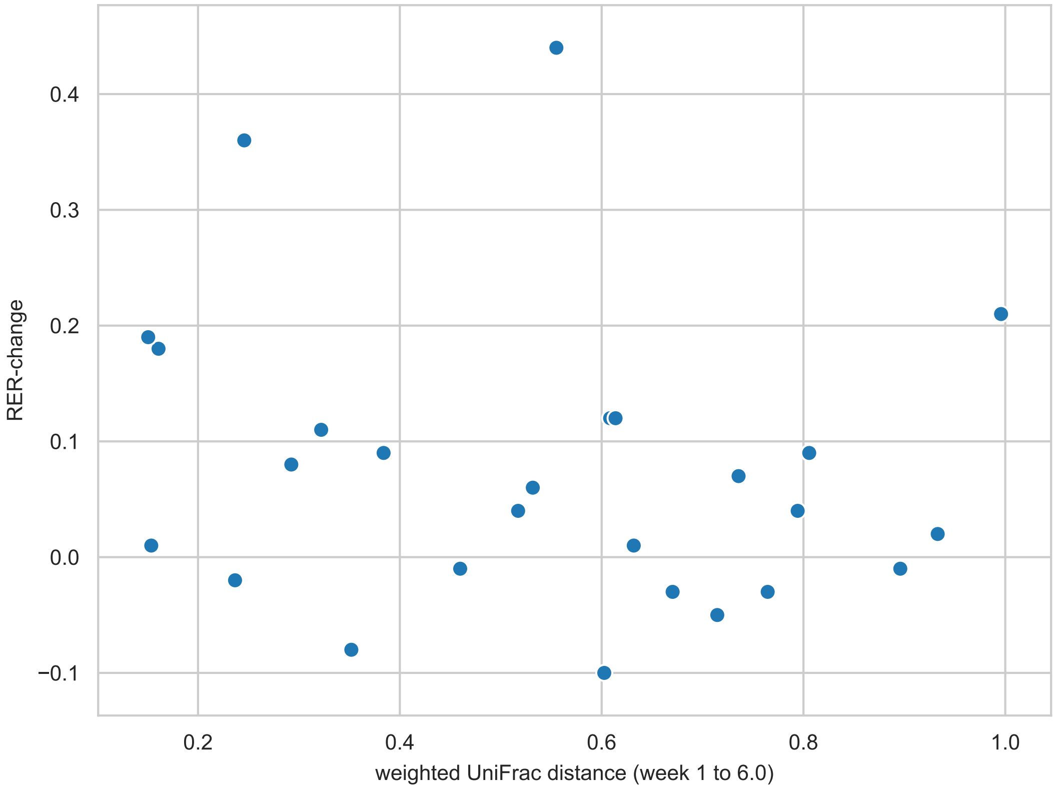

RER-change
0.4
0.3
0.2
0.1
0.0
−0.1
0.2
0.4
0.6
0.8
1.0
weighted UniFrac distance (week 1 to 6.0)

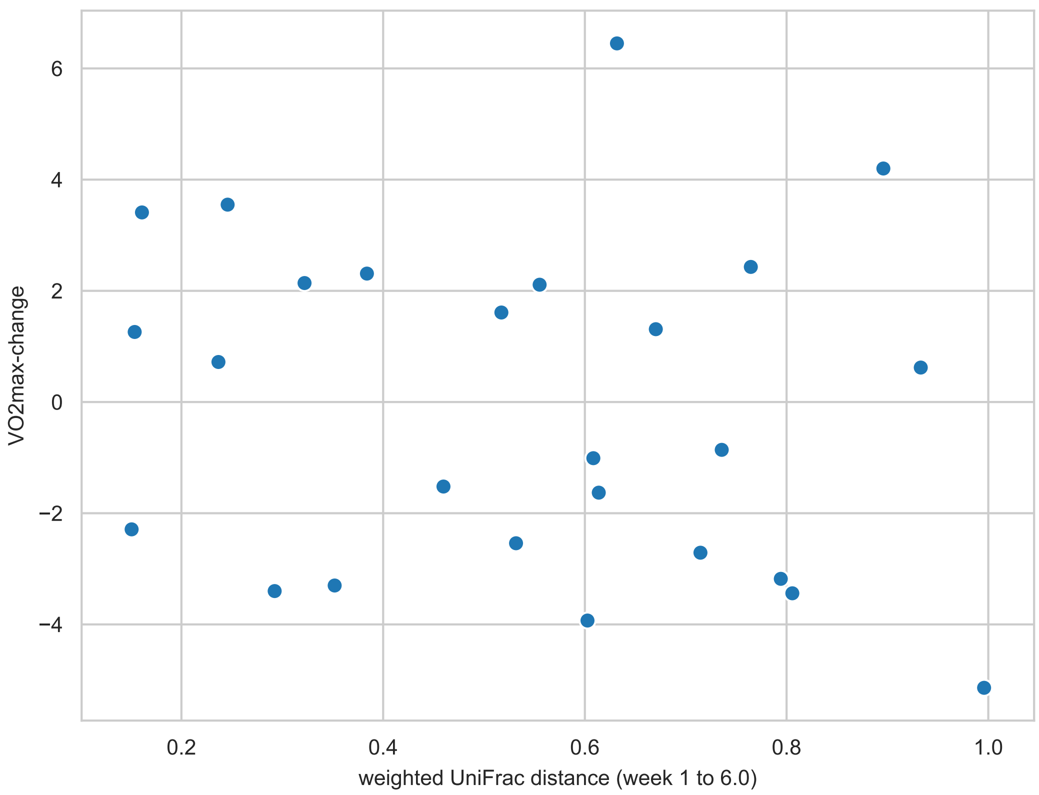
VO2max-change
weighted UniFrac distance (week 1 to 6.0)
6
4
2
0
−2
−4
0.2
0.4
0.6
0.8
1.0

project	distance	performance metric	Spearman rho
exmp2	unweighted UniFrac distance (week 1 to 5.0)	bench-press-change	0.197446571
exmp2	unweighted UniFrac distance (week 1 to 5.0)	row-change	-0.140413728
exmp2	unweighted UniFrac distance (week 1 to 5.0)	3RM-squat-change	0.118600728
exmp2	weighted UniFrac distance (week 1 to 5.0)	bench-press-change	0.092141733
exmp2	weighted UniFrac distance (week 1 to 5.0)	row-change	0.258944796
exmp2	weighted UniFrac distance (week 1 to 5.0)	3RM-squat-change	0.264156168
exmp2	Bray-Curtis distance (week 1 to 5.0)	bench-press-change	0.242577216
exmp2	Bray-Curtis distance (week 1 to 5.0)	row-change	0.047412428
exmp2	Bray-Curtis distance (week 1 to 5.0)	3RM-squat-change	0.271344091
exmp2	Jaccard distance (week 1 to 5.0)	bench-press-change	0.188044354
exmp2	Jaccard distance (week 1 to 5.0)	row-change	-0.072942196
exmp2	Jaccard distance (week 1 to 5.0)	3RM-squat-change	0.136570536

Spearman p-value	sample size	Spearman q-value
0.480587596	15	0.99470216
0.617690452	15	0.99687758
0.673769414	15	0.99687758
0.743970749	15	0.99687758
0.351379275	15	0.9915111
0.341419303	15	0.9915111
0.383694033	15	0.9915111
0.866748467	15	0.99687758
0.327945833	15	0.9915111
0.502133114	15	0.99470216
0.796145426	15	0.99687758
0.627440321	15	0.99687758

project	distance	performance metric	Spearman rho
exmp2	unweighted UniFrac distance (week 1 to 6.0)	bench-press-change	0.166426567
exmp2	unweighted UniFrac distance (week 1 to 6.0)	row-change	0.119544142
exmp2	unweighted UniFrac distance (week 1 to 6.0)	3RM-squat-change	0.183632363
exmp2	weighted UniFrac distance (week 1 to 6.0)	bench-press-change	0.178146748
exmp2	weighted UniFrac distance (week 1 to 6.0)	row-change	0.036088797
exmp2	weighted UniFrac distance (week 1 to 6.0)	3RM-squat-change	0.429212994
exmp2	Bray-Curtis distance (week 1 to 6.0)	bench-press-change	0.407862291
exmp2	Bray-Curtis distance (week 1 to 6.0)	row-change	-0.045110997
exmp2	Bray-Curtis distance (week 1 to 6.0)	3RM-squat-change	0.418150803
exmp2	Jaccard distance (week 1 to 6.0)	bench-press-change	0.24377976
exmp2	Jaccard distance (week 1 to 6.0)	row-change	0.166910688
exmp2	Jaccard distance (week 1 to 6.0)	3RM-squat-change	0.298679145

Spearman p-value	sample size	Spearman q-value
0.569597804	14	0.99491378
0.683967172	14	0.99491378
0.52973547	14	0.99491378
0.542309464	14	0.99491378
0.902518227	14	0.99491378
0.125645364	14	0.80035823
0.147714619	14	0.80169728
0.878296292	14	0.99491378
0.136781404	14	0.80169728
0.400976013	14	0.98342118
0.56845946	14	0.99491378
0.299603591	14	0.95944026

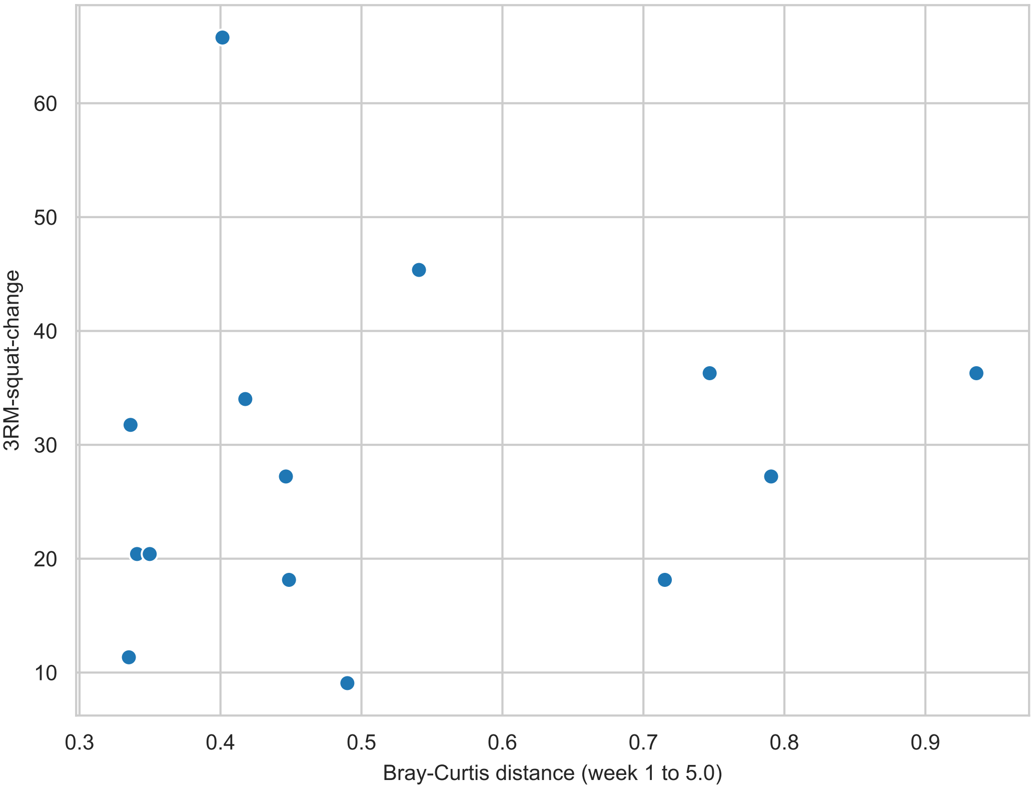

3RM-squat-change
60
50
40
30
20
10
0.3
0.4
0.5
0.6
0.7
0.8
0.9
Bray-Curtis distance (week 1 to 5.0)

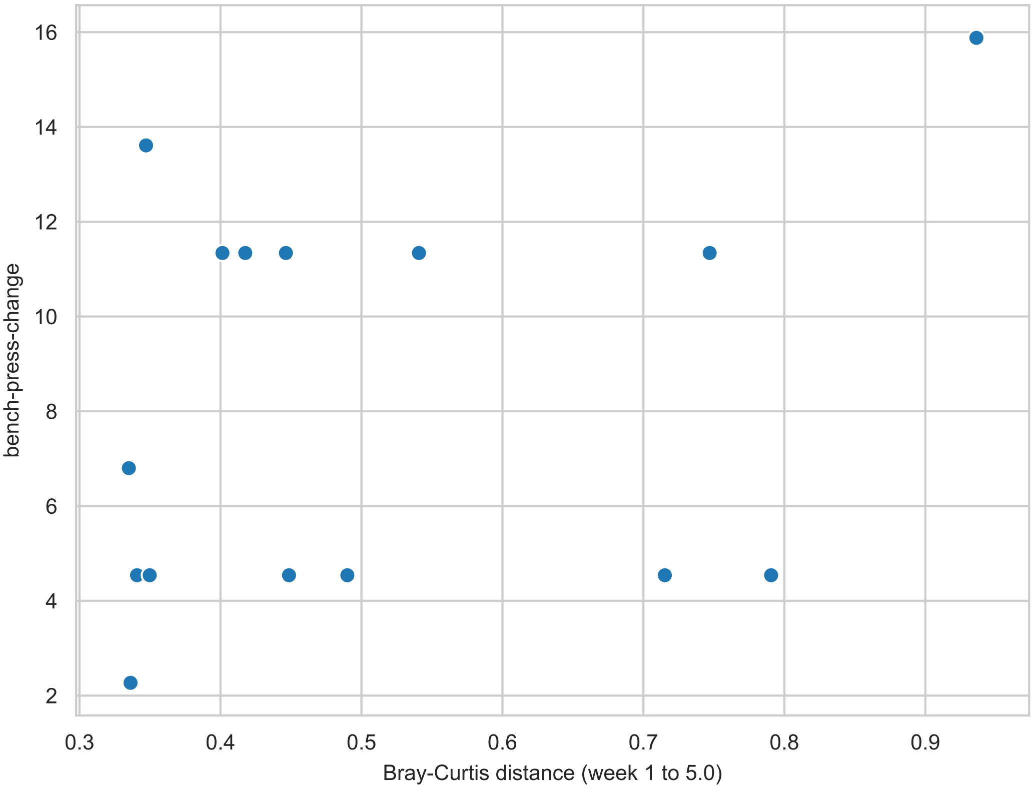
bench-press-change
16
14
12
10
8
6
4
2
0.3
0.4
0.5
0.6
0.7
0.8
0.9
Bray-Curtis distance (week 1 to 5.0)

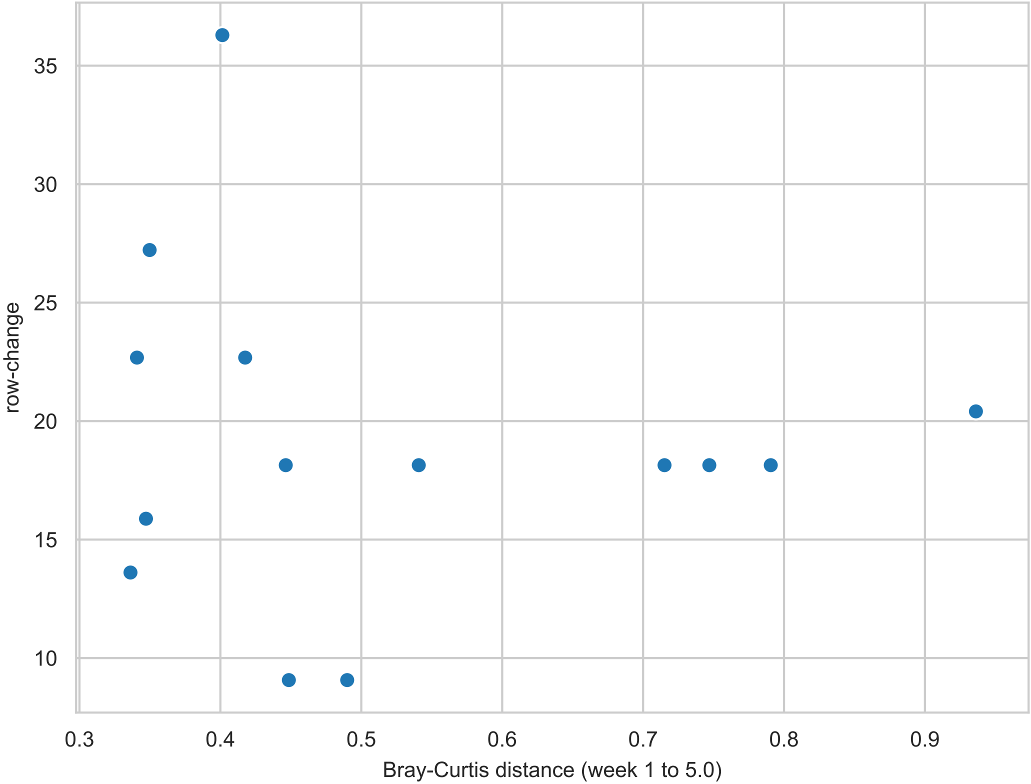
35
30
25
20
15
10
row-change
0.3
0.4
0.5
0.6
0.7
0.8
0.9
Bray-Curtis distance (week 1 to 5.0)

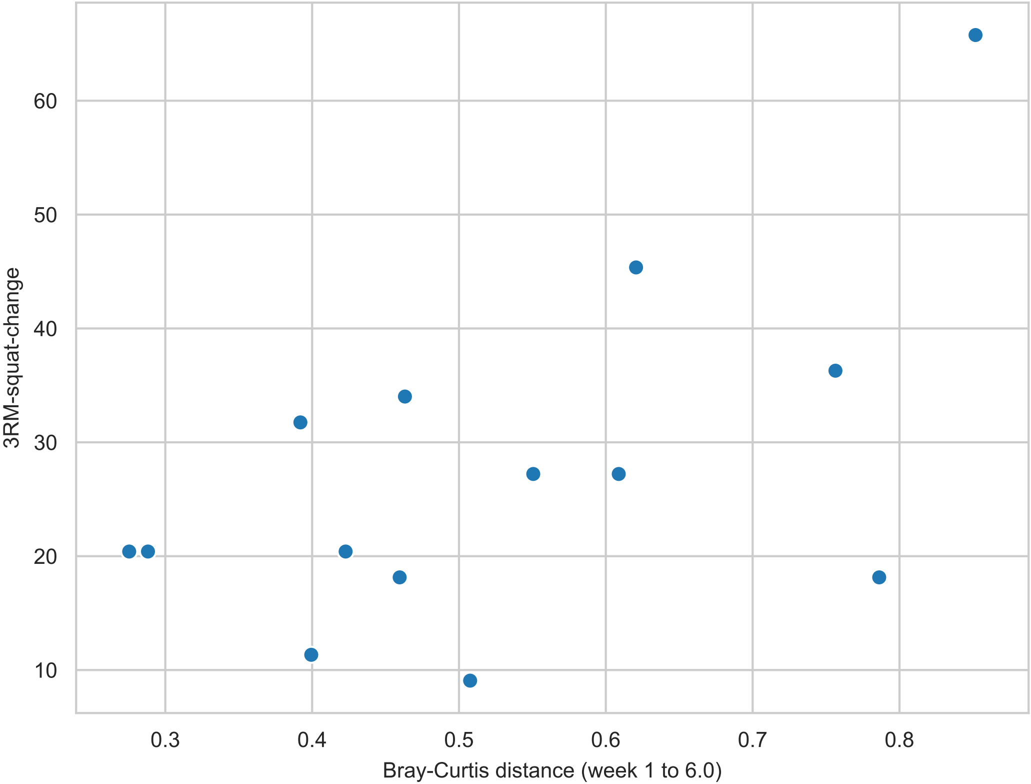

60
50
40
30
20
10
3RM-squat-change
0.3
0.4
0.5
0.6
0.7
0.8
Bray-Curtis distance (week 1 to 6.0)

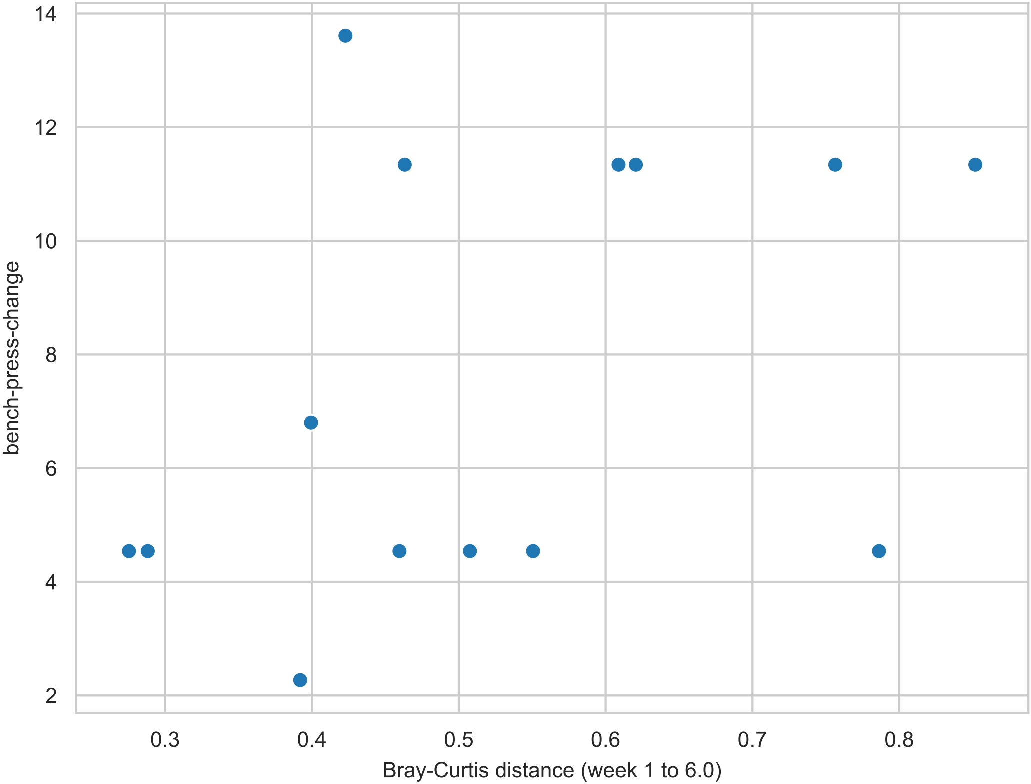

14
12
10
8
6
4
2
bench-press-change
0.3
0.4
0.5
0.6
0.7
0.8
Bray-Curtis distance (week 1 to 6.0)

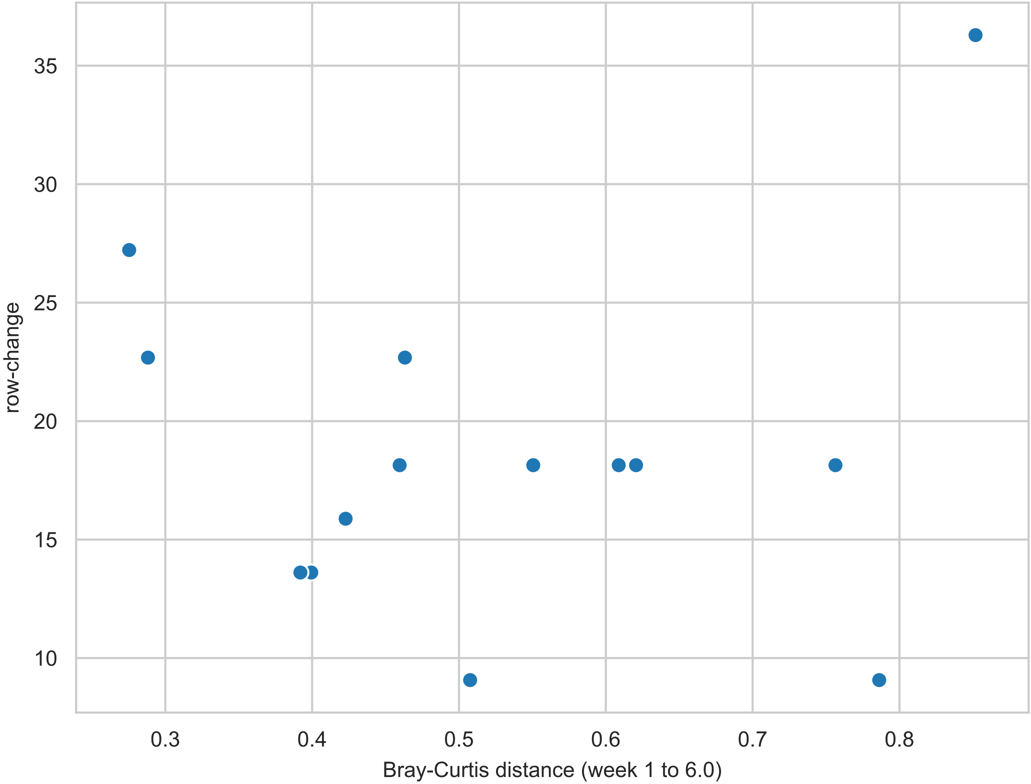

35
30
25
20
15
10
row-change
0.3
0.4
0.5
0.6
0.7
0.8
Bray-Curtis distance (week 1 to 6.0)

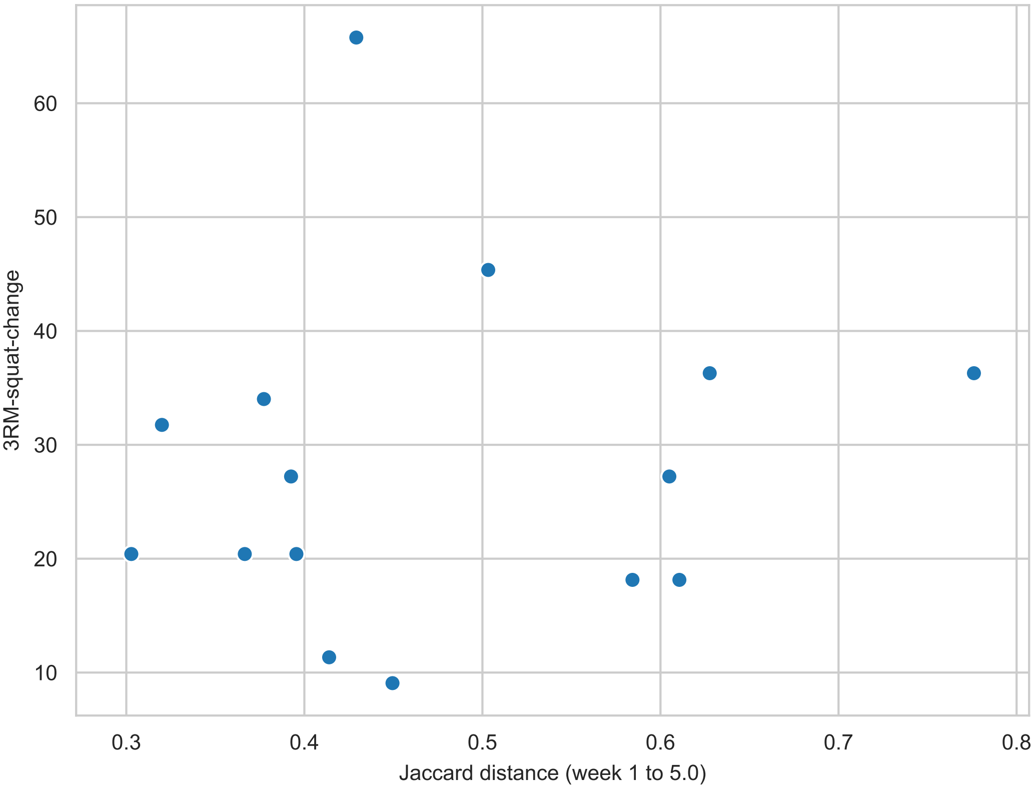
3RM-squat-change
60
50
40
30
20
10
0.3
0.4
0.5
0.6
0.7
0.8
Jaccard distance (week 1 to 5.0)

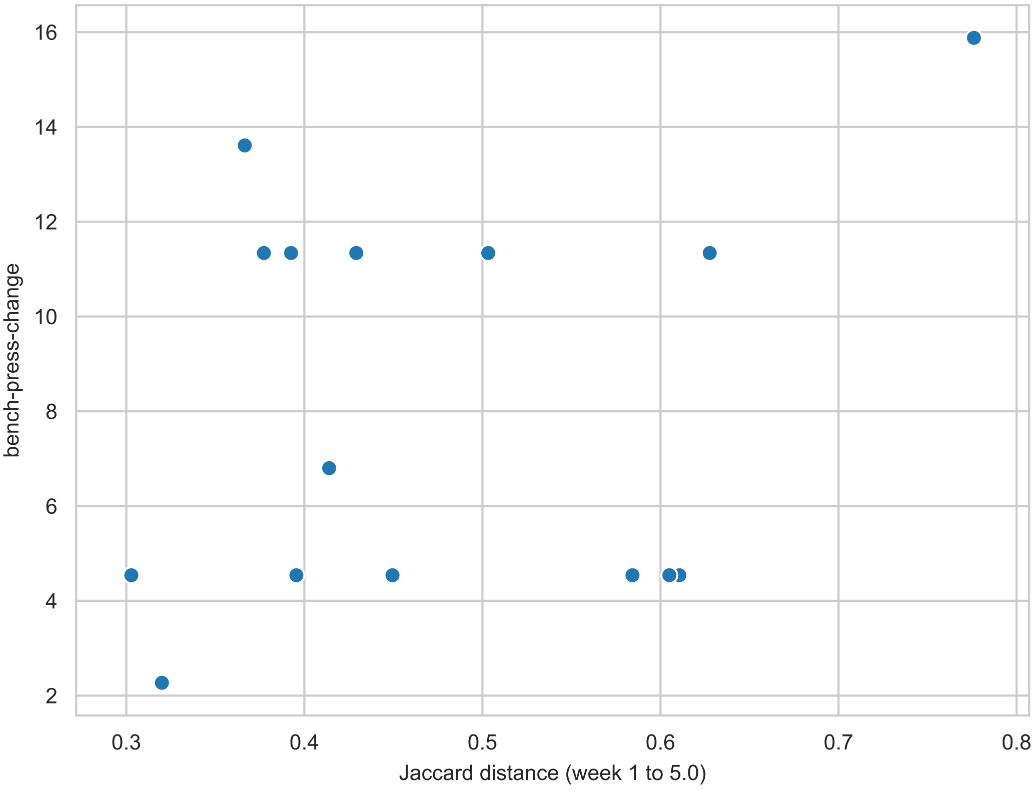

16
14
12
10
8
6
4
2
bench-press-change
0.3
0.4
0.5
0.6
0.7
0.8
Jaccard distance (week 1 to 5.0)

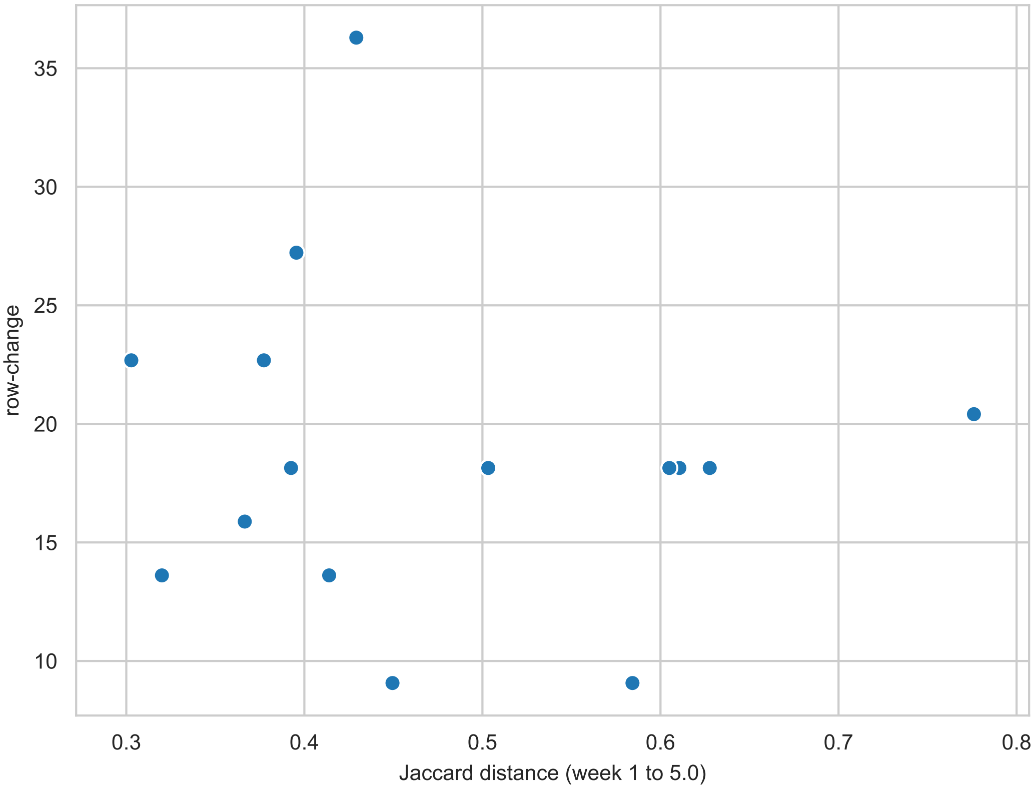
35
30
25
20
15
10
row-change
0.3
0.4
0.5
0.6
0.7
0.8
Jaccard distance (week 1 to 5.0)

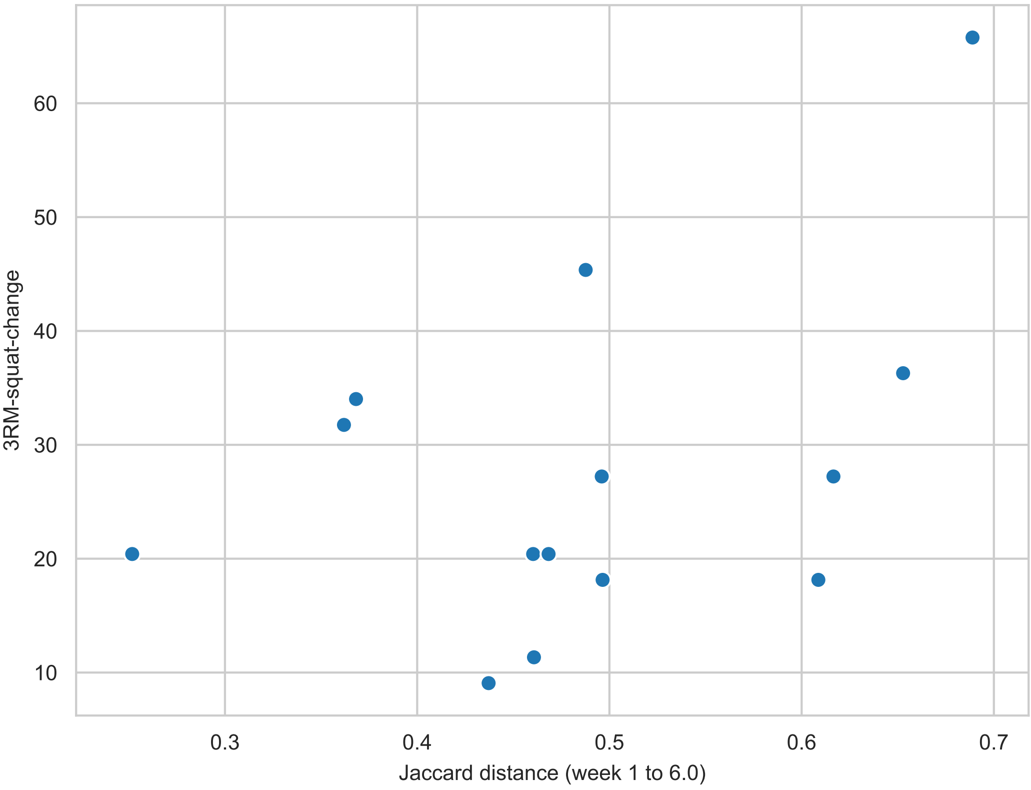
3RM-squat-change
60
50
40
30
20
10
0.3
0.4
0.5
0.6
0.7
Jaccard distance (week 1 to 6.0)

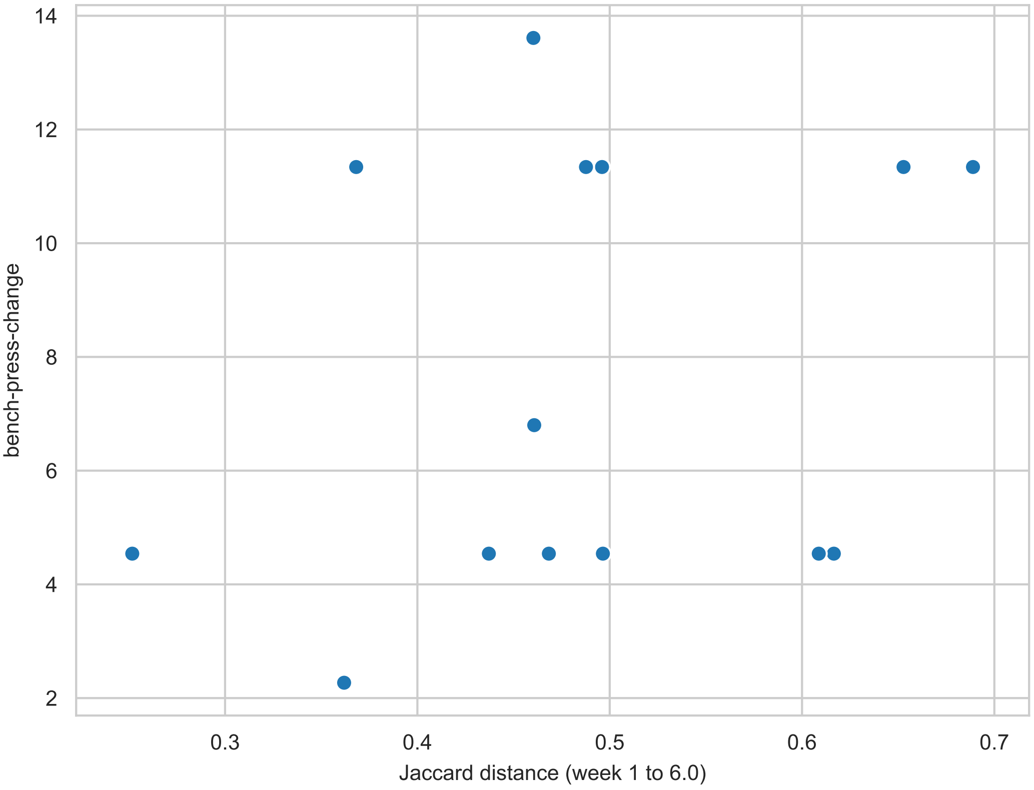

bench-press-change
14
12
10
8
6
4
2
0.3
0.4
0.5
0.6
0.7
Jaccard distance (week 1 to 6.0)

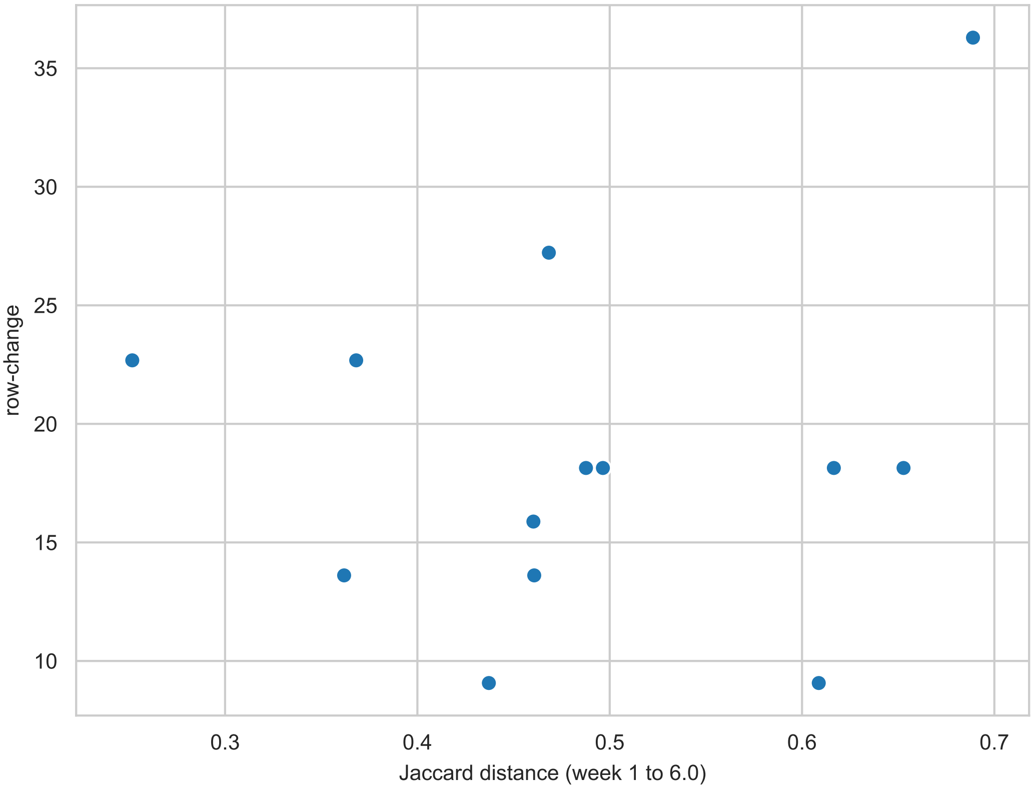
35
30
25
20
15
10
row-change
0.3
0.4
0.5
0.6
0.7
Jaccard distance (week 1 to 6.0)

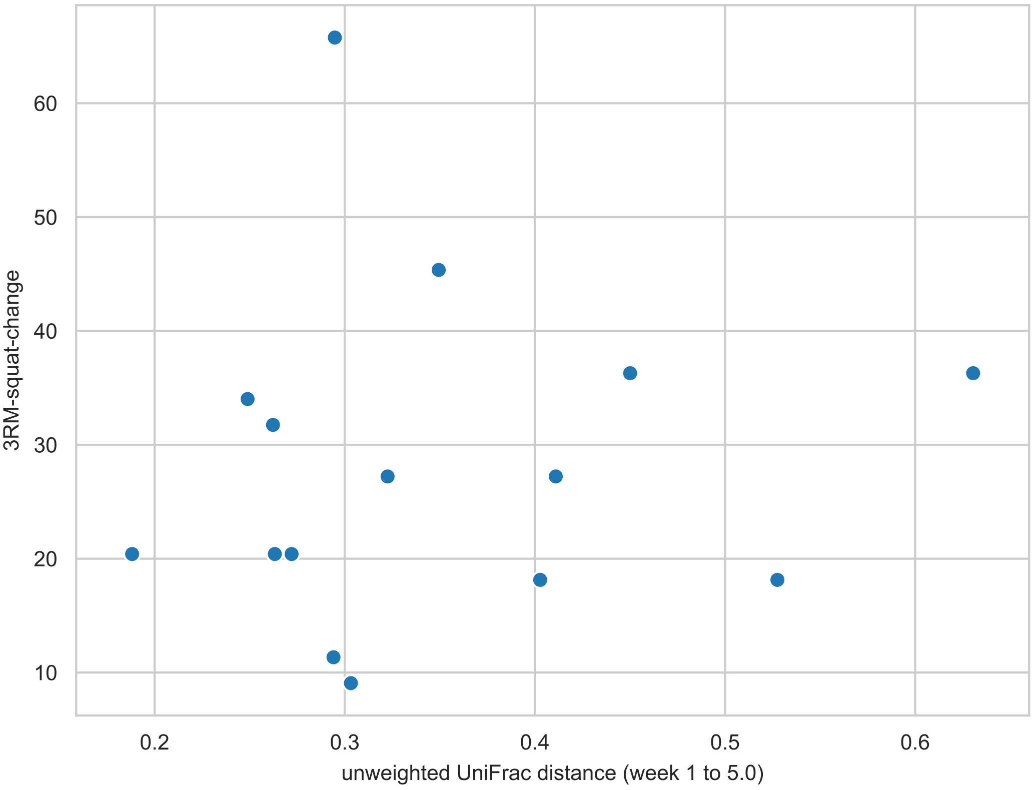

60
50
40
30
20
10
3RM-squat-change
0.2
0.3
0.4
0.5
0.6
unweighted UniFrac distance (week 1 to 5.0)

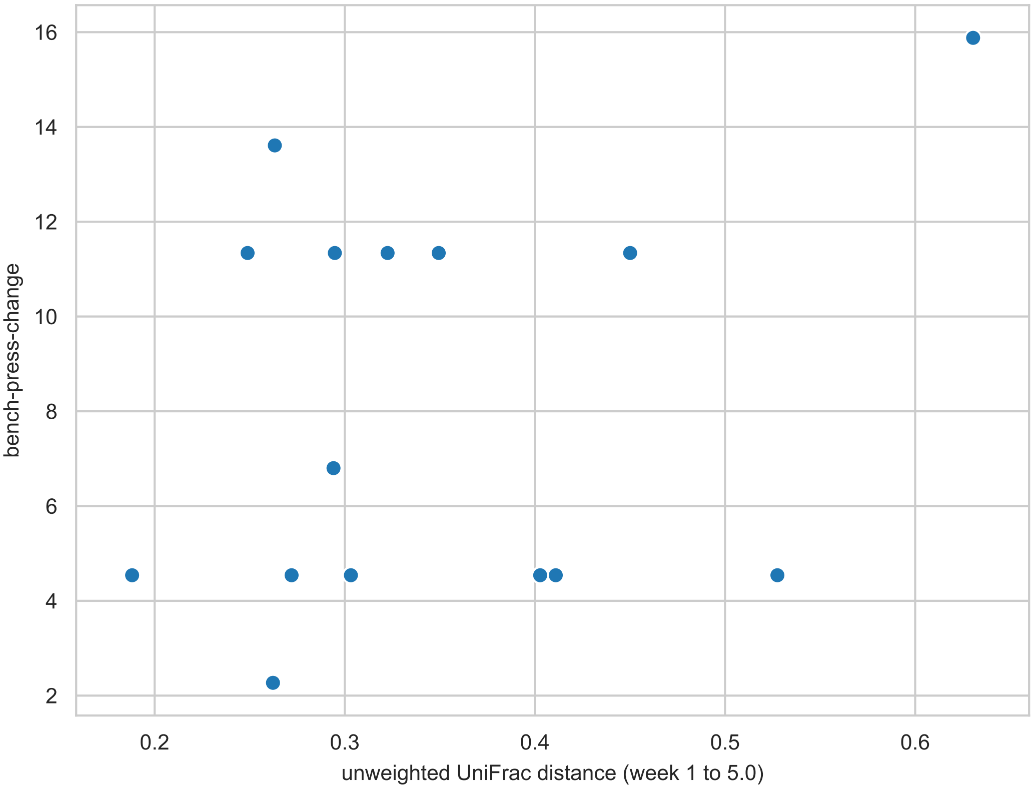
16
14
12
10
8
6
4
2
bench-press-change
0.2
0.3
0.4
0.5
0.6
unweighted UniFrac distance (week 1 to 5.0)

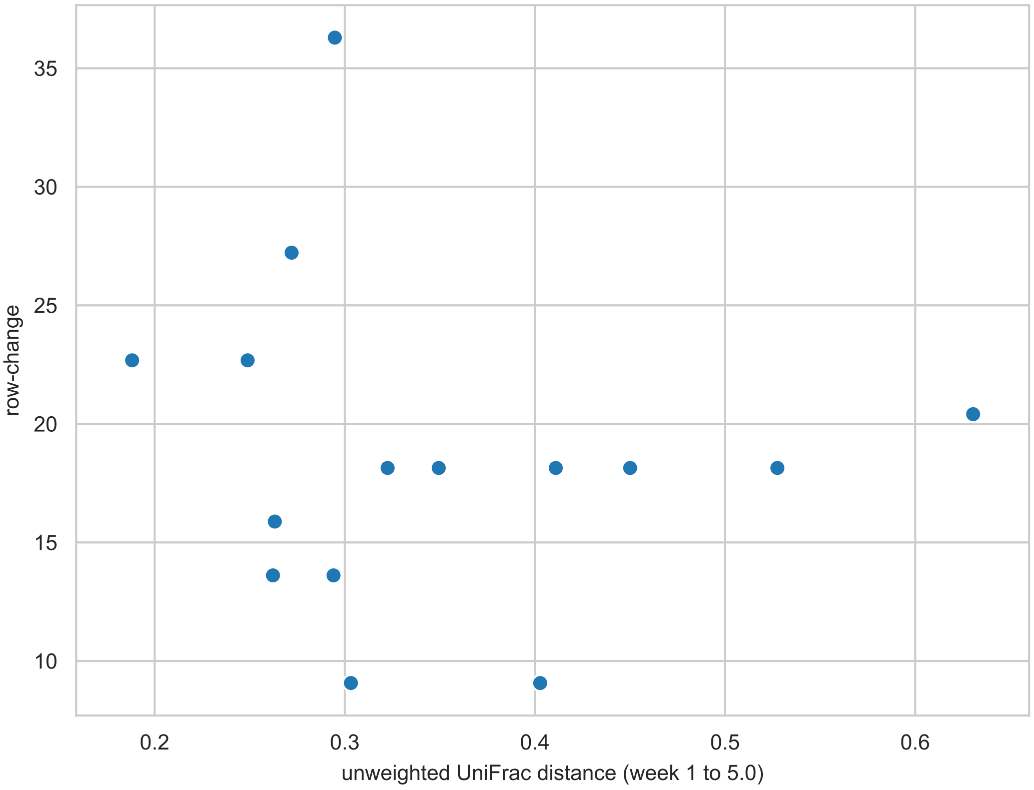
35
30
25
20
15
10
row-change
0.2
0.3
0.4
0.5
0.6
unweighted UniFrac distance (week 1 to 5.0)

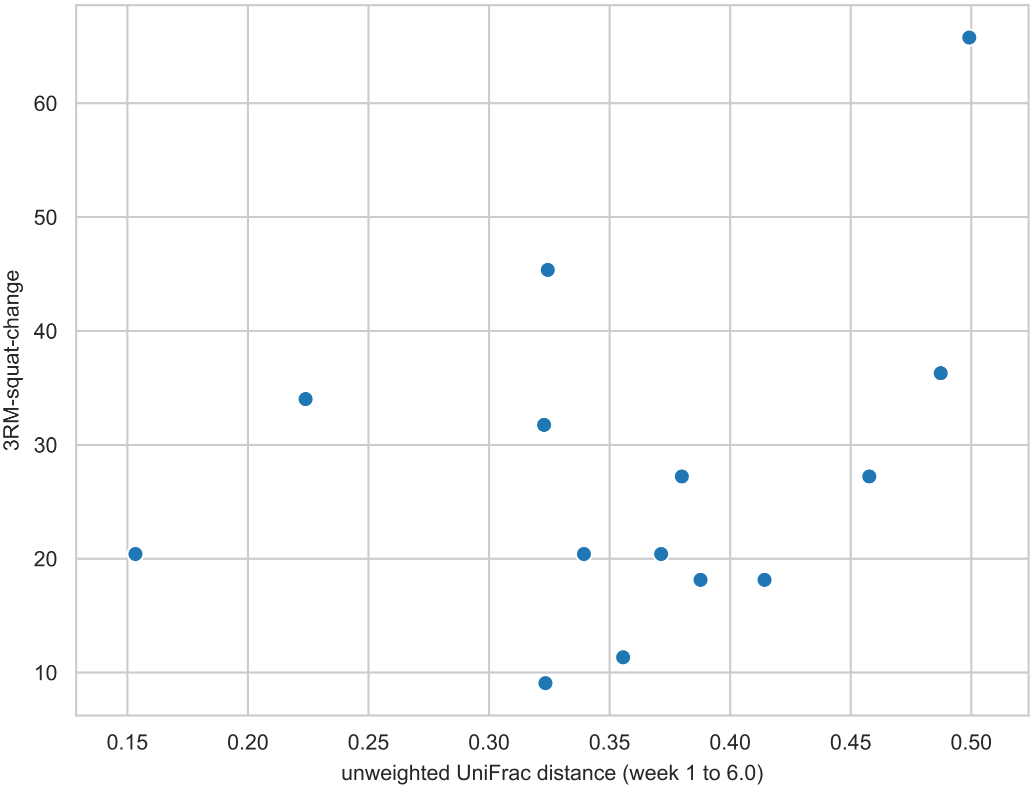

60
50
40
30
20
10
3RM-squat-change
0.15
0.20
0.25
0.30
0.35
0.40
0.45
0.50
unweighted UniFrac distance (week 1 to 6.0)

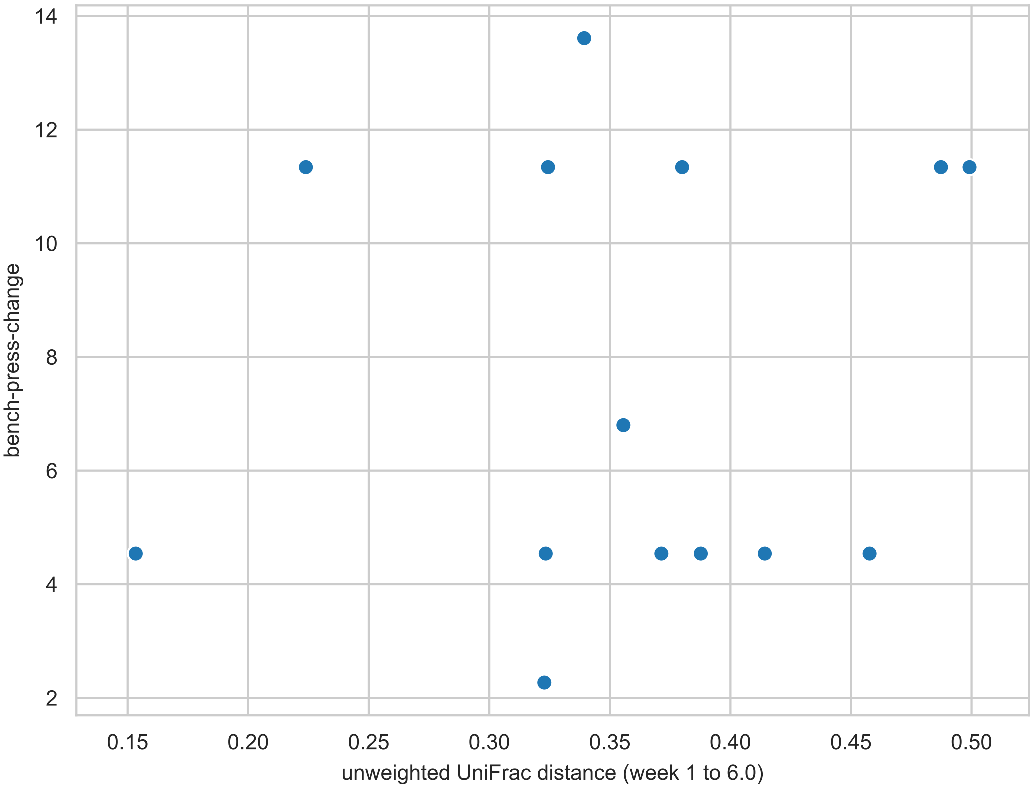

bench-press-change
14
12
10
8
6
4
2
0.15
0.20
0.25
0.30
0.35
0.40
0.45
0.50
unweighted UniFrac distance (week 1 to 6.0)

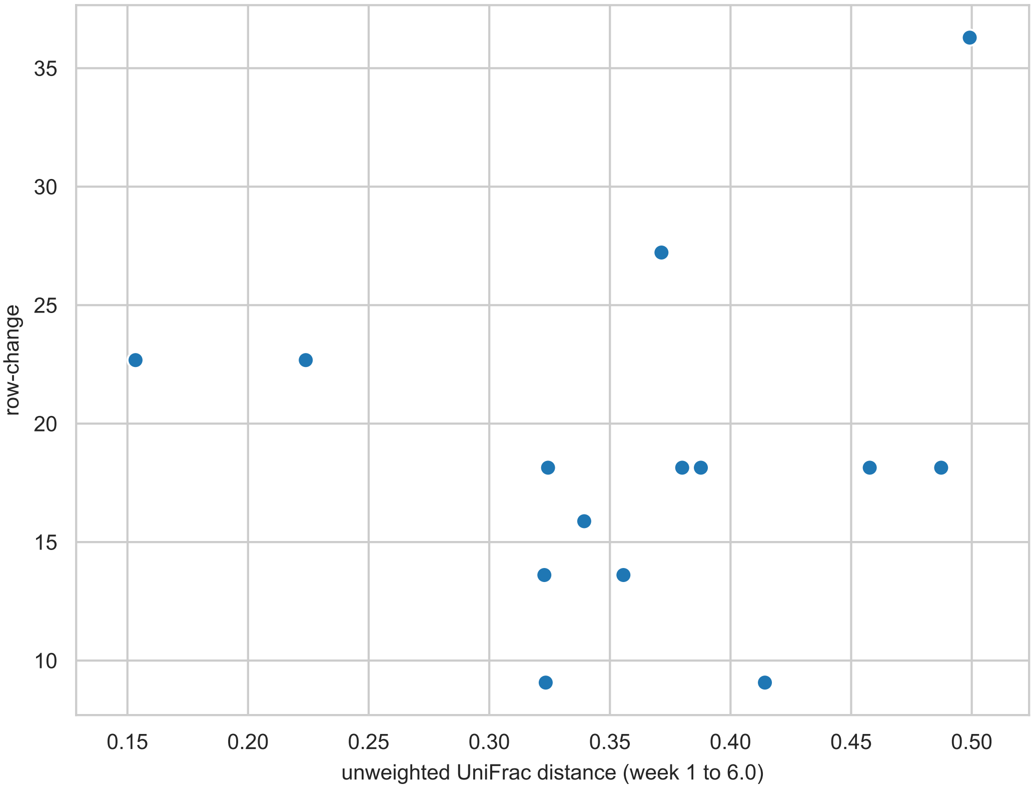
35
30
25
20
15
10
row-change
0.15
0.20
0.25
0.30
0.35
0.40
0.45
0.50
unweighted UniFrac distance (week 1 to 6.0)

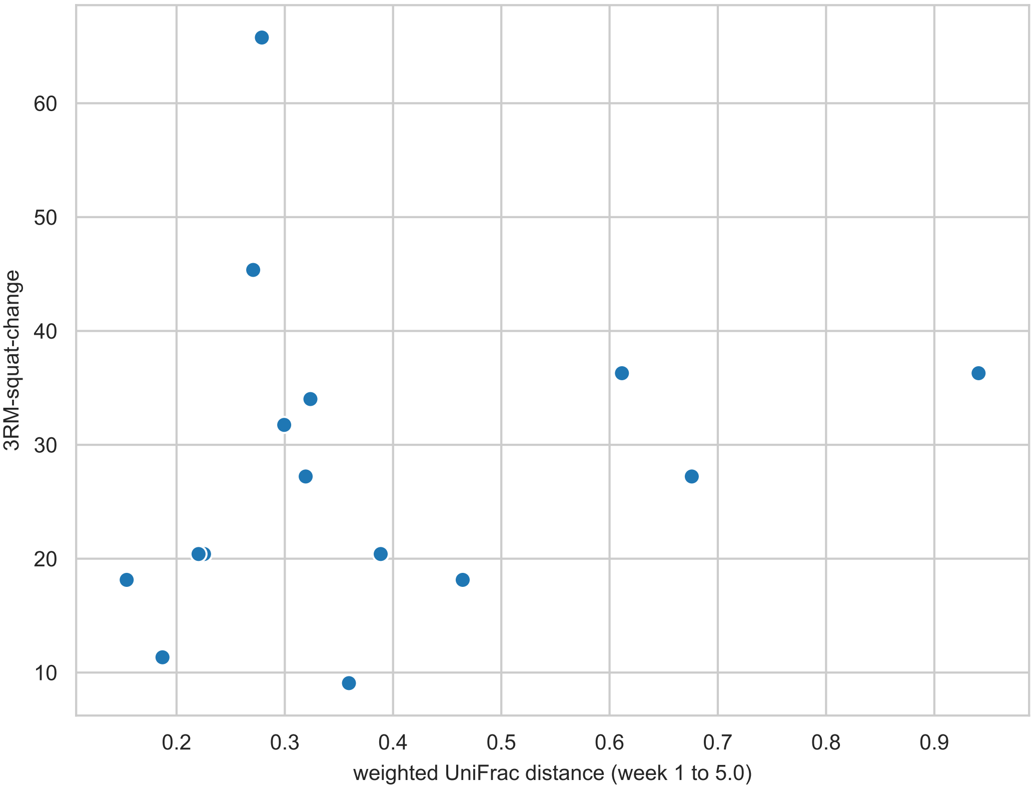
3RM-squat-change
10
20
30
40
50
60
0.2
0.3
0.4
0.5
0.6
0.7
0.8
0.9
weighted UniFrac distance (week 1 to 5.0)

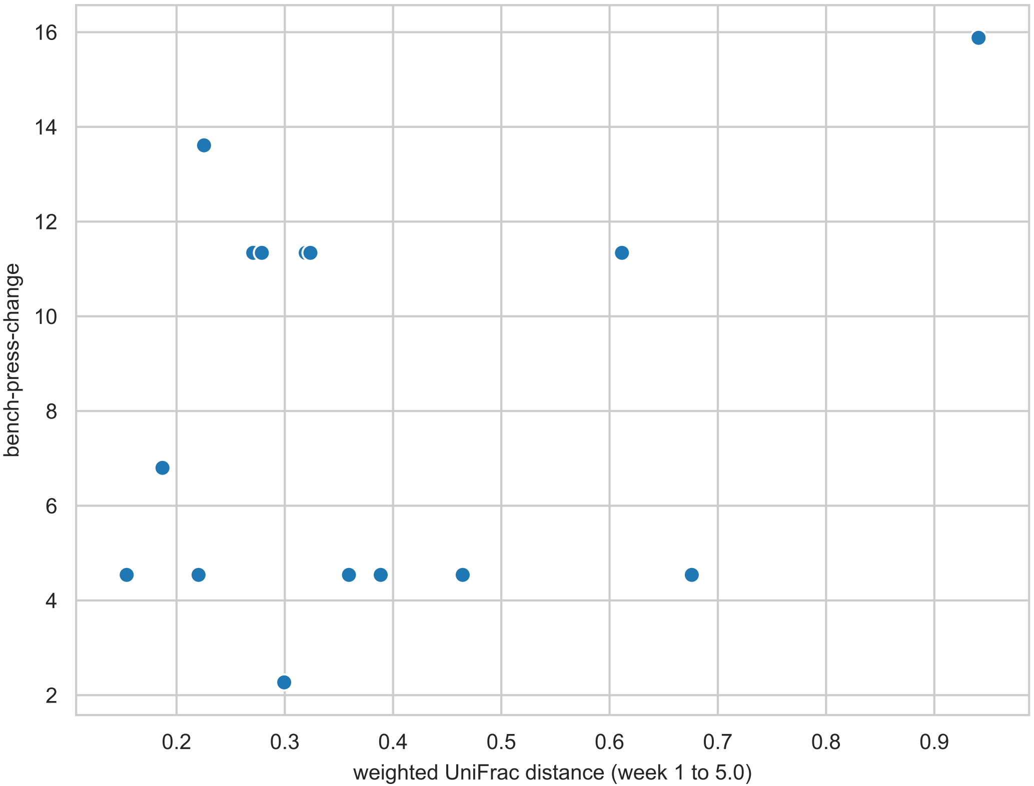

bench-press-change
weighted UniFrac distance (week 1 to 5.0)
16
14
12
10
8
6
4
2
0.2
0.3
0.4
0.5
0.6
0.7
0.8
0.9

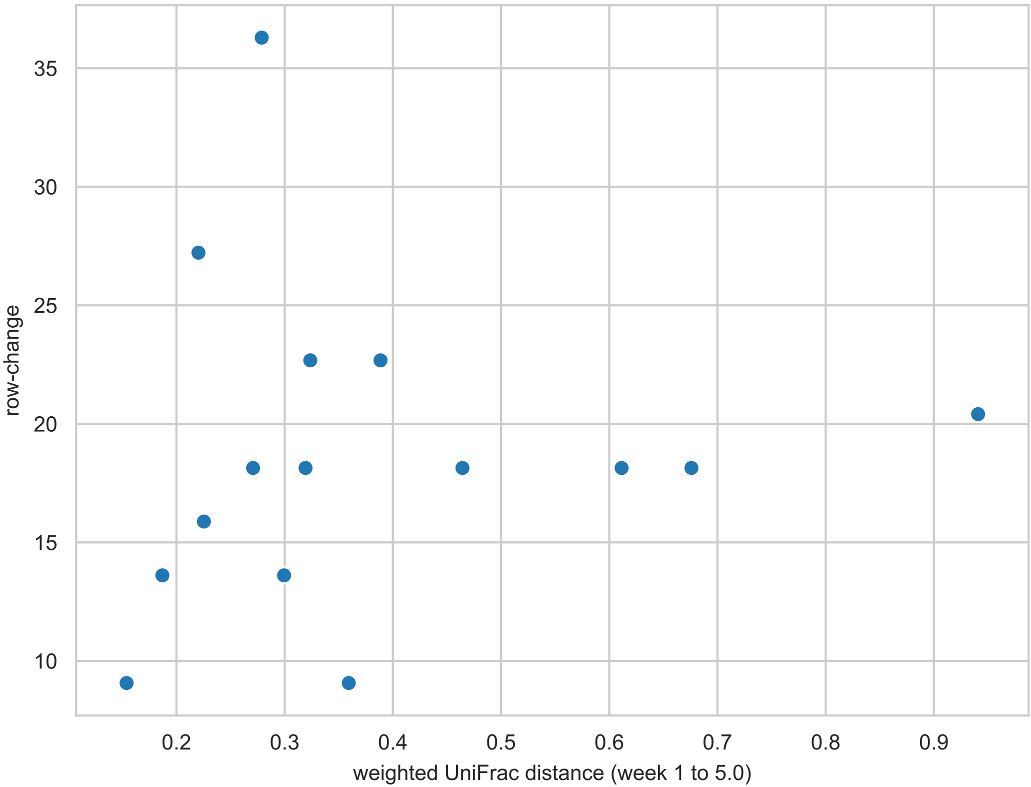

35
30
25
20
15
10
row-change
0.2
0.3
0.4
0.5
0.6
0.7
0.8
0.9
weighted UniFrac distance (week 1 to 5.0)

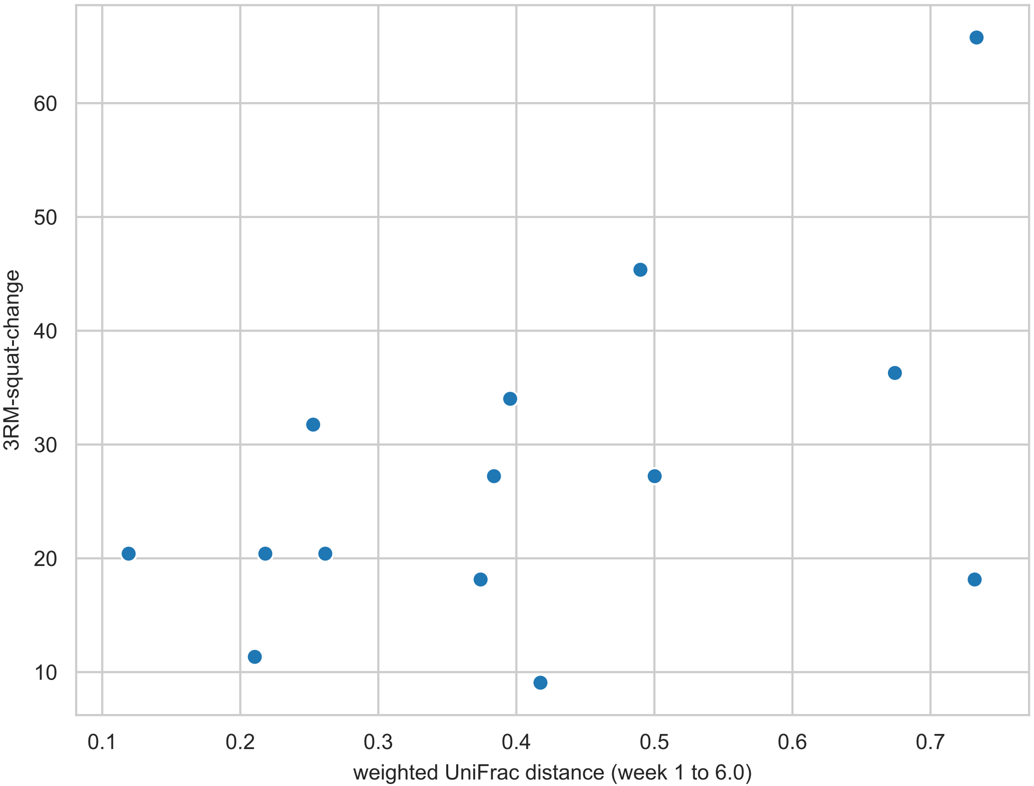
3RM-squat-change
weighted UniFrac distance (week 1 to 6.0)
10
20
30
40
50
60
0.1
0.2
0.3
0.4
0.5
0.6
0.7

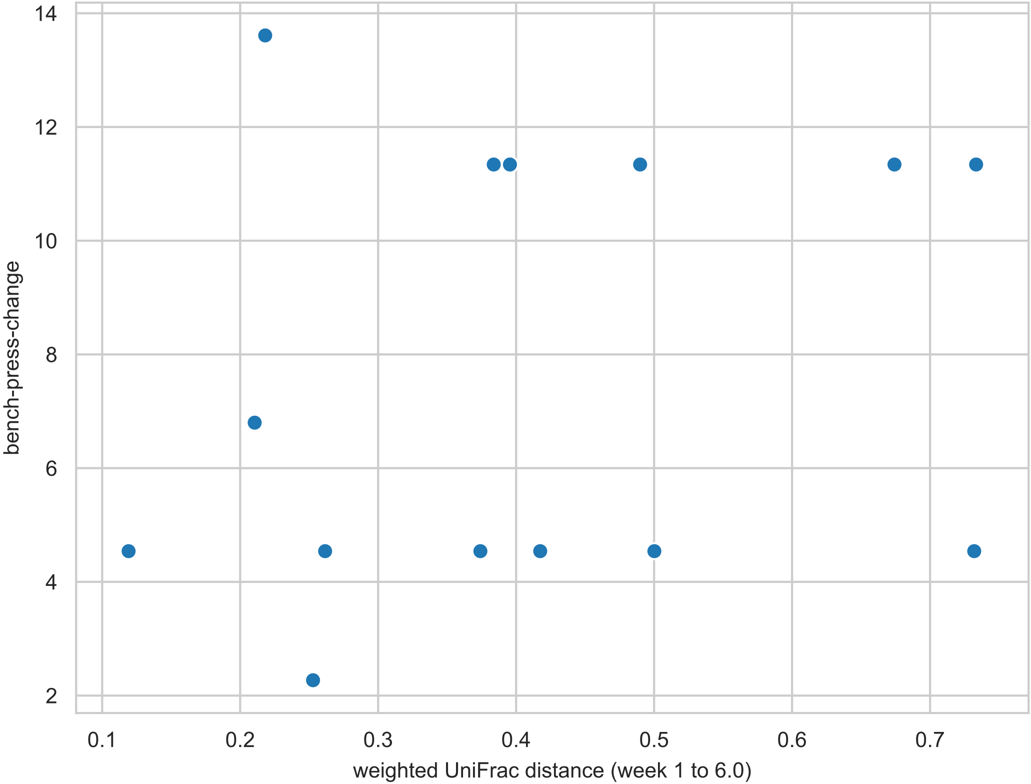

14
12
10
8
6
4
2
bench-press-change
0.1
0.2
0.3
0.4
0.5
0.6
0.7
weighted UniFrac distance (week 1 to 6.0)

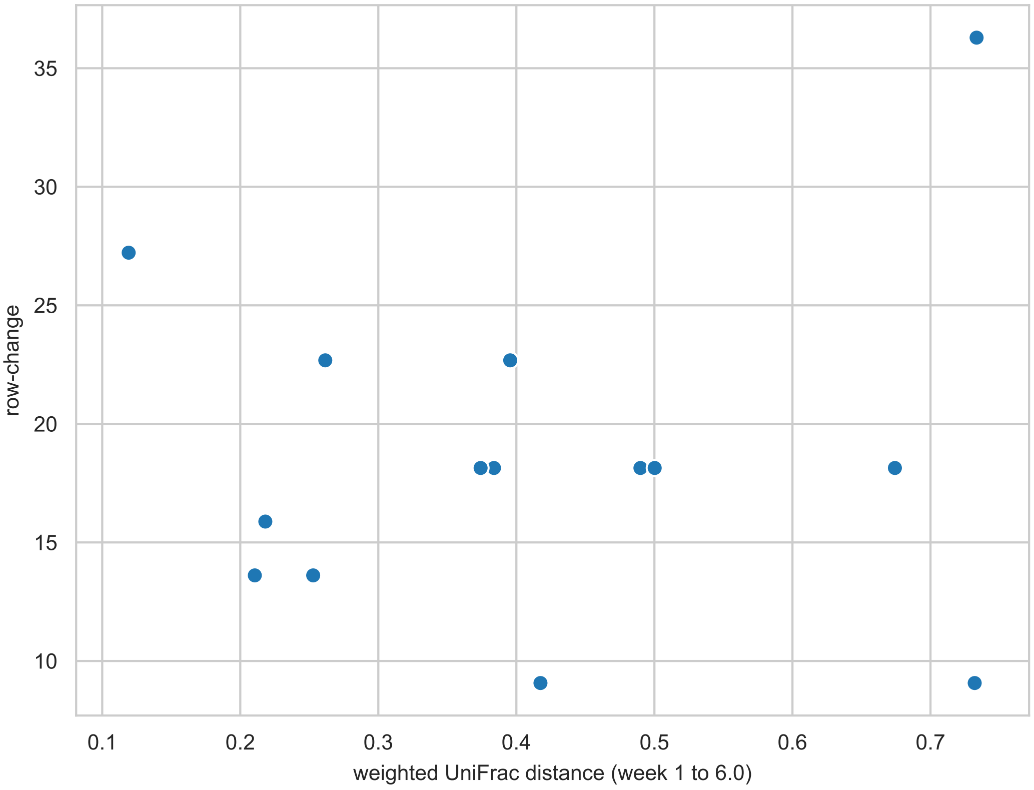

35
30
25
20
15
10
row-change
0.1
0.2
0.3
0.4
0.5
0.6
0.7
weighted UniFrac distance (week 1 to 6.0)
