# Supplementary material for: Impact of Different Exercise Modalities on the Human Gut Microbiome
Source: Sports (Basel). 2021 Jan 21;9(2):14. doi: 10.3390/sports9020014 (PMC7909775; doi:10.3390/sports9020014)
Supplement: Supplementary file 1 [file sports-09-00014-s001.zip › Supplementary Materials/Spreadsheet S4.docx]

| **Supplementary Table 1.** EXMP-CRE subjects’ VO_2max_ classifications by sex over study period. | | | | | | |
| --- | --- | --- | --- | --- | --- | --- |
|  | **Female Participants (n = 21)** | |  | **Male Participants (n = 7)** | |  |
| **Classification** | **Pre** | **Post** |  | **Pre** | **Post** |  |
| Poor | 10 | 11 |  | 5 | 6 |  |
| Fair | 8 | 9 |  | 2 | 1 |  |
| Good | 3 | 1 |  |  |  |  |
| Excellent |  |  |  |  |  |  |
| Superior |  |  |  |  |  |  |
| All VO_2max_ classifications reflect those listed by Gibson, Wagner, and Heyward [30]. Subjects’ age groups were removed for readability and VO_2max_ classifications are listed only by biological sex. | | | | | |  |

**Supplemental Digital Content 4:** EXMP-CRE subjects’ VO_2max_ classifications by sex over study period.
