# Supplementary material for: Impact of Different Exercise Modalities on the Human Gut Microbiome
Source: Sports (Basel). 2021 Jan 21;9(2):14. doi: 10.3390/sports9020014 (PMC7909775; doi:10.3390/sports9020014)
Supplement: Supplementary file 1 [file sports-09-00014-s001.zip › Supplementary Materials/Spreadsheet S5.docx]

| **Supplemental Table 2.** EXMP-RTE subjects’ relative strength categories by sex over study period. | | | | | |
| --- | --- | --- | --- | --- | --- |
|  | **Bench Press ^a^** | | | | |
|  | **Female Participants (n = 17)** | |  | **Male Participants (n = 11)** | |
| **Classification** | **Pre** | **Post** |  | **Pre** | **Post** |
| Well Below Average |  |  |  | 6 | 2 |
| Below Average | 1 |  |  | 2 | 3 |
| Average | 1 |  |  | 2 | 4 |
| Above Average | 7 |  |  | 1 | 2 |
| Well Above Average | 8 | 17 |  |  |  |
|  | **Squat ^b^** | | | | |
| **Classification** | **Pre** | **Post** |  | **Pre** | **Post** |
| Poor | 16 | 7 |  | 9 | 3 |
| Fair | 1 | 1 |  | 1 | 3 |
| Average |  | 2 |  | 1 | 4 |
| Good |  | 4 |  |  |  |
| Excellent |  | 3 |  |  | 1 |
| Subjects’ age groups were removed for readability and relative strength classifications are listed only by biological sex. ^a^ All bench press normative values and classifications reflect those listed by Gibson, Wagner, and Heyward [30]. ^b^ All squat normative values and classifications reflect those listed by from Hoffman [31]. | | | | | |

**Supplemental Digital Content 5:** EXMP-RTE subjects’ relative strength categories by sex over study period.
