# Supplementary material for: Impact of Different Exercise Modalities on the Human Gut Microbiome
Source: Sports (Basel). 2021 Jan 21;9(2):14. doi: 10.3390/sports9020014 (PMC7909775; doi:10.3390/sports9020014)
Supplement: Supplementary file 1 [file Spreadsheet S2.pdf › Supplementary Materials/Spreadsheet S2.pdf]

Participant ID: ______________

EXMP-II Lifting Card (Full Body)

Date:	Date			Date			Date			Date		
**Full Body**	Pred.	Reps	Wt.	Pred.	Reps	Wt.	Pred.	Reps	Wt.	Pred.	Reps	Wt.
1. Squats												
1A. Wall Ball Toss												
2. Bent Over Rows												
2A. Hamstring Curls												
3. Bench Press												
3A. Ball Roll-Ins												
4. Romanian Deadlifts												
4A. Triceps												

Participant ID: ______________

EXMP-II Lifting Card (Lower Body)

Date:	Date			Date			Date			Date		
**Lower Body**	Pred.	Reps	Wt.	Pred.	Reps	Wt.	Pred.	Reps	Wt.	Pred.	Reps	Wt.
1. Sumo Squats												
1A. Rotating Side Planks 6 ea. side												
2. Glute Bridges w/ Dumbbell on Hips												
2A. Plank Sidesteps 12 ea. side												
3. Deadlifts												
3A. Stability Ball Pass												
4. Side Lunges 6 ea. side												
4A. Box Jumps 12” or 16”												

Participant ID: ______________

EXMP-II Lifting Card (Upper Body)

Date:	Date			Date			Date			Date		
**Upper Body**	Pred.	Reps	Wt.	Pred.	Reps	Wt.	Pred.	Reps	Wt.	Pred.	Reps	Wt.
1. Bench Press												
1A. Supermans												
2. Bent Over Rows												
2A. Running Man Sit-Ups												
3. Triceps												
3A. Reverse Flies												
4. Lat. Pull-Ups / Assisted												
4A. Plank w/ Lateral Knee In												
